# Structural Diversity and Bioactivities of Mangrove-Derived Fungal Polyketids (2020–2025)

**DOI:** 10.3390/md23120474

**Published:** 2025-12-11

**Authors:** Miao Yu, Caijuan Zheng, Guangjin Zheng, Haofu Dai, Qiang Wang

**Affiliations:** 1 College of Chemical and Material, Guangxi Minzu Normal University, Chongzuo 532200, China; yumiaonpc@126.com (M.Y.); 15778098407@163.com (G.Z.); 2 College of Chemistry and Chemical Engineering, Hainan Normal University, Haikou 571158, China; caijuan2002@163.com; 3 Hainan Key Laboratory of Research and Development of Natural Product from Li Folk Medicine, Institute of Tropical Bioscience and Biotechnology, Chinese Academy of Tropical Agricultural Sciences, Haikou 571101, China

**Keywords:** mangrove-derived fungi, polyketides, biological activity

## Abstract

Mangrove forests represent a complex ecosystem inhabiting tropical and subtropical intertidal zones, harboring diverse microbial communities including fungi, actinomycetes, bacteria, cyanobacteria, algae, and protozoa. Among these communities, mangrove-derived fungi, as the second-largest ecological group of marine fungi, not only play essential roles in establishing and sustaining this biosphere but also serve as an important source of structurally unique and biologically active secondary metabolites. This review systematically summarizes research progress on metabolites isolated from mangrove-derived fungi and their associated bioactivities over the recent five years (2020–2025). Emphasis is placed on 457 metabolites documented in 97 selected publications, with a focus on the biological activities and distinctive chemical diversity of these secondary metabolites. This review provides an important reference for the research status of secondary metabolites isolated from mangrove-derived fungi and the lead compounds worthy of further development, and reveals that mangrove-derived fungi have important medicinal values and are worthy of further development.

## 1. Introduction

Fungi are widely distributed in nature and are recognized as an important source of natural products due to their abundant secondary metabolites and biosynthetic gene clusters [1]. The unique environmental conditions of mangrove ecosystems, such as periodic tidal inundation, contribute to the production of structurally novel and biologically active metabolites by mangrove-derived fungi. To date, more than 1500 secondary metabolites have been isolated from these fungi, over 40% of which exhibit biological activities such as anti-inflammatory and antimicrobial effects. As a result, mangrove-derived fungi have become a significant source of lead compounds for marine drug discovery [2].

Polyketides represent a major structural class among the secondary metabolites of mangrove fungi, characterized by diverse structural features and a wide range of biological activities. According to Cox et al., polyketide synthases (PKS) play a crucial role in the biosynthesis of polyketides. Variations in the extent of chain elongation, chain length, and modification levels during biosynthesis contribute to their structural diversity and abundance [3,4]. Polyketides derived from mangrove fungi such as chromones, quinones, naphthalenes, and phenols exhibit significant potential in drug development and agricultural biocontrol due to their unique chemical structures and broad pharmacological activities. As noted by Hang et al. [5], these compounds have wide applications in pharmaceutical production, representative drugs containing polyketide structures include griseofulvin [6] and lovastatin [7].

Based on this research background, this review summarizes 457 polyketide compounds reported between January 2020 and February 2025. These compounds are categorized into seven structural types: coumarins and isocoumarins, chromones, xanthones, quinones, lactones, azaphilones, and others. Among them, five compounds represent novel skeletons, while 201 compounds exhibit various biological activities, including cytotoxicity, antimicrobial, anti-inflammatory, and enzyme inhibitory effects. This paper systematically outlines the structural characteristics and biological activities of polyketides from mangrove-derived fungi, as well as their potential contributions to future medicinal and agricultural natural product development, thereby providing a theoretical reference for further research and resource utilization of mangrove fungal polyketides.

## 2. Secondary Metabolites from Mangrove-Derived Fungal Polyketids and Their Bioactivities

### 2.1. Coumarins and Isocoumarins

**Figure 1:**
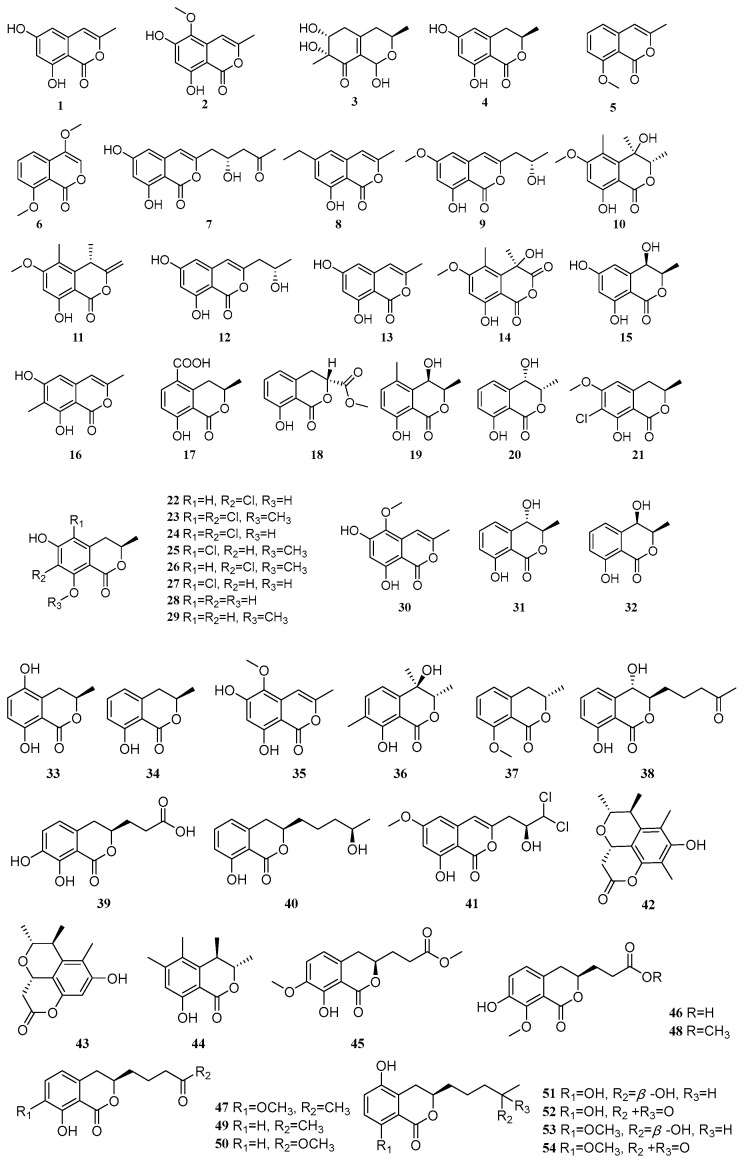

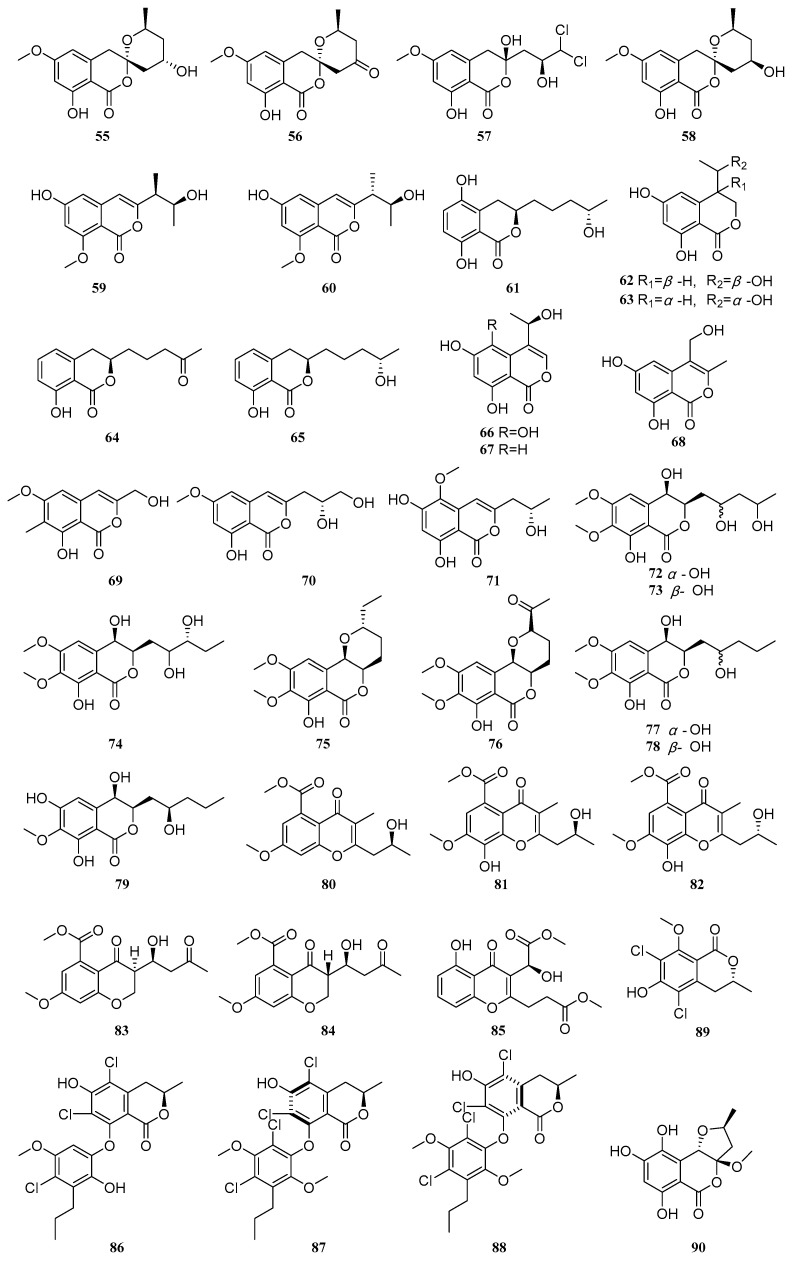

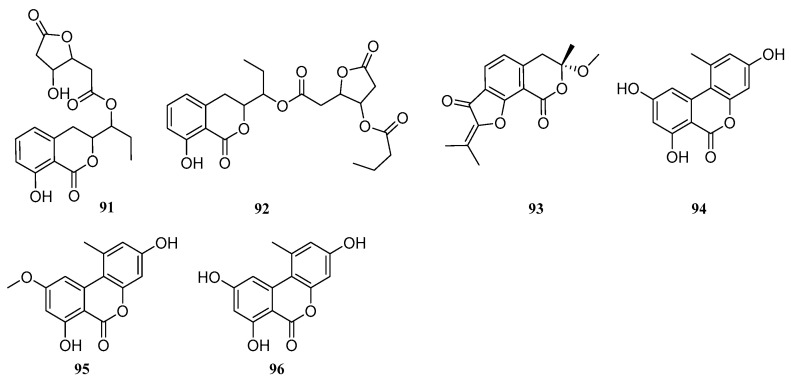
Coumarins and isocoumarins in polyketide compounds of mangrove fungi.

Coumarins and isocoumarins represent a significant class of bioactive compounds widely distributed in nature. A total of 96 coumarin and isocoumarin compounds have been isolated from mangrove-derived fungi, with their structures illustrated in Figure 1. Among these, 37 compounds exhibit biological activities such as anti-inflammatory, antimicrobial, and antioxidant effects.

Two compounds, 3-methyl-6,8-dihydroxyisocoumarin (**1**) and 6,8-dihydroxy-5-methoxy-3-methyl-1*H*-isochromen-1-one (**2**), were isolated from the mangrove fungus *Penicillium* sp. SCSIO 41411. Activity screening revealed that compounds **1** and **2** exhibited weak inhibitory activity against PDE4, with inhibition rates of 27.42% and 27.39%, respectively, at a concentration of 10 µM [8]. Five compounds, fusaraisochromenone (**3**), 3*R*-3,4-dihydro-6,8-dihydroxy-3-methylisocoumarin (**4**), 2-acetyl-7-methoxybenzofuran (**5**), 4,8-dimethoxy-1*H*-isochromen-1-one (**6**), and (+)-citreoisocoumarin (**7**), were isolated from the mangrove endophytic fungus *Daldinia eschscholzii* MCZ-18. Compounds **3** and **5** exhibited broad-spectrum inhibitory activity against five pathogenic strains, *Enterococcus faecalis*, methicillin-resistant *Staphylococcus aureus* (MRSA), *Escherichia coli*, *Pseudomonas aeruginosa*, and *Candida albicans*, with IC_50_ values ranging from 6.25 to 50 µM. Compound **7** demonstrated significant inhibitory activity against MRSA with an IC_50_ value of 6.25 µM. Preliminary structure-activity relationship studies suggested that the oxygen-containing heterocyclic structure may enhance the compound’s effect against *P. aeruginosa*, *E. faecalis*, MRSA, and *E. coli*. Furthermore, the position of -OH and -OCH_3_ substitutions on the benzene ring or lactone moiety of the isocoumarin backbone appears to confer selectivity towards different pathogenic bacteria [9]. Seven compounds, 8-hydroxy-6-methoxy-3-methyl-1*H*-isochromen-1-one (**8**), (*S*)-8-hydroxy-3-(2-hydroxypropyl)-6-methoxy-1*H*-isochromen-1-one (**9**), (3*S*,4*R*)-4,8-dihydroxy-6-methoxy-3,4,5-trimethylisochroman-1-one (**10**), (*S*)-8-hydroxy-6-methoxy-4,5-dimethyl-3-methyleneisochroman-1-one (**11**), (*S*)-6,8-dihydroxy-3-(2-hydroxypropyl)-1*H*-isochromen-1-one (**12**), 6,8-dihydroxy-3-methyl-1*H*-isochromen-1-one (**13**), and 4,8-dihydroxy-6-methoxy-4,5-dimethyl-3-methyleneisochroman-1-one (**14**), were isolated from the mangrove sediment-derived fungus *Roussoella* sp. SCSIO 41427 [10]. Two compounds, 4,6-dihydroxymellein (**15**) and similanpyrone B (**16**), were isolated from the mangrove-derived fungus *Talaromyces* sp. WHUF0362 [11]. One compound, (-)-mellein-5-carboxylic acid (**17**), was isolated from the mangrove-derived fungus TBRC-BCC 64093 [12]. One compound, tenuissimasatin (**18**), was isolated from the mangrove-derived fungus *Mollisia* sp. SCSIO41409 [13]. In another study, Cai et al. isolated two compounds, (3*R*,4*R*)-cis-4-hydroxy-5-methylmellein (**19**) and 3*S*,4*R*-4-hydroxy-mellein (**20**), from the mangrove-derived fungus *Phomopsis* sp. HYP11. Compounds **19** and **20** demonstrated significant antioxidant activity, with IC_50_ values of 0.09 mM and 0.17 mM, respectively, which were stronger than that of the positive control Trolox (IC_50_ = 0.29 mM) [14].

One compound, 7-chloro-6-methoxymellein (**21**), was isolated from the mangrove endophytic fungus *Aspergillus* sp. GXNU-A9 [15]. One new natural product, 7-chloro-3,4-dihydro-6,8-dihydroxy-3-methylisocoumarine (**22**), along with seven known compounds, (*S*)-5,7-dichloro-6-methoxy-2-methyl-2,3-dihydrobenzofuran-4-carboxylic acid (**23**), pericochlorosin A (**24**), palmaerones F-G (**25**–**26**), 5-chloro-6-hydroxymellein (**27**), (*R*)-6-hydroxymellein (**28**), and 3-methyl-6-hydroxy-8-methoxy-3,4-dihydroisocoumarin (**29**), were isolated from the mangrove endophytic fungus *Amorosia* sp. SCSIO 41026. At non-toxic concentrations, compounds **22**, **23**, **27**, and **29** inhibited the production of nitric oxide and pro-inflammatory cytokines in lipopolysaccharide (LPS)-induced RAW264.7 macrophages. Specifically, these compounds suppressed both the mRNA expression and release of the pro-inflammatory cytokines IL-6 and TNF-*α*. Further in vivo studies demonstrated that compound **27** alleviated pathological lung injury in LPS-treated mice and protected RAW264.7 macrophages from LPS-induced inflammatory responses by inhibiting the PI3K/AKT pathway [16].

One compound, 6,8-dihydroxy-5-methoxy-3-methyl-1*H*-isochromen-1-one (**30**), was isolated from the mangrove endophytic fungus *Phyllosticta capitalensis*. Compound **30** exhibited weak inhibitory activity against *Pseudomonas aeruginosa* and *Staphylococcus aureus*, with a MIC value of 225 μM [17]. Four compounds, (-)-trans-axial-4-hydroxymellein (**31**), (-)-cis-equatorial-4-hydroxymellein (**32**), 4,8-dihydroxy-3-methylisochroman-1-one (5-hydroxymellein) (**33**), and mellein (**34**), were isolated from the mangrove fungus *Lasiodiplodia theobromae*. Compounds **31**–**34** showed significant inhibitory activity against *Trypanosoma brucei*, with IC_50_ values ranging from 1.20 to 4.10 μM [18].

Xu et al. isolated one compound, 6,8-dihydroxy-5-methoxy-3-methyl-1*H*-isochromen-1-one (**35**), from the mangrove endophytic fungus *Aspergillus fumigatus* HQD24 [19]. In another study, Xu et al. isolated one new compound, pestalotiopisorin B (**36**), and one known compound, (*R*)-(-)-mellein methyl ether (**37**), from the mangrove-derived fungus *Pestalotiopsis* sp. HHL101. Compound **36** exhibited antibacterial activity against *Escherichia coli* and *Pseudomonas aeruginosa*, with IC_50_ values of 56.31 and 225.23 μM, respectively [20]. Three compounds, aspergillumarin C (**38**), (3*R*)-(7,8-dihydroxy-1-oxoisochroman-3-yl)propanoic acid (**39**), and aspergillumarin B (**40**), were isolated from the mangrove endophytic fungus *Talaromyces* sp. SCNU-F0041 [21]. One compound, dichlorodiaportin (**41**), was isolated from the mangrove sediment-derived fungus *Trichoderma harzianum* SCSIO 41051 [22].

One new compound, cladosporin E (**42**), along with two known compounds, cladosporin C (**43**) and decarboxydihydrocitrinone (**44**), were isolated from the mangrove sediment-derived fungus *Talaromyces* sp. SCSIO 41428. Compound **43** exhibited significant inhibitory activity against prostate cancer cells PC-3 and 22Rv1, with IC_50_ values of 6.10 and 9.25 µM, respectively [23]. A new compound, penicimarin N (**45**), was isolated from the mangrove endophytic fungus *Penicillium* sp. TGM112. Compound **45** demonstrated strong antioxidant activity with an IC_50_ value of 1.0 µM, and also showed moderate inhibitory activity against α-glucosidase with an IC_50_ value of 620 µM [24]. Two new compounds, penicimarins L-M (**46**–**47**), and seven known compounds, peniciisocoumarin E (**48**), aspergillumarin A (**49**), penicimarin I (**50**), peniciisocoumarin F (**51**), penicilloxalone B (**52**), penicimarin G (**53**), and penicimarin H (**54**), were isolated from the mangrove endophytic fungus *Penicillium* sp. MGP11. All compounds, except **50** and **51**, exhibited antioxidant activity, with IC_50_ values ranging from 4.6 to 40.5 µM. The activity of compound **53** (IC_50_ = 4.6 µM) was stronger than that of the positive control Trolox (IC_50_ = 12.9 µM). Compounds **50**, **53**, and **54** showed *α*-glucosidase inhibitory activity, with IC_50_ values of 776.5, 683.7, and 868.7 µM, respectively, compared to the positive control acarbose (IC_50_ = 313.9 µM) [25]. Two new compounds, penicillol A (**55**) and penicillol B (**56**), along with two known compounds, dichlorodiaportal (**57**) and citreoviranol (**58**), were isolated from the mangrove endophytic fungus *Penicillium* sp. BJR-P2. Compound **56** inhibited NO production in LPS-induced RAW264.7 cells with an IC_50_ value of 12.0 µM, which was stronger than the positive control indomethacin (IC_50_ = 35.8 µM). Molecular docking studies were conducted to further investigate the mechanism by which compound **56** inhibits NO production. The results indicated that compound **56** interacts with the active site of inducible nitric oxide synthase (iNOS) by forming multiple characteristic hydrogen bonds. In contrast, the carbonyl group at position 4′ in compound **55** differs from the hydroxyl group in **56**, resulting in a distinct conformation for **55** that prevents the formation of hydrogen bonds with key amino acid residues in the iNOS active region, thereby explaining its lack of inhibitory activity [26]. Four new compounds, hypoxymarins A-D (**59**–**62**), and six known compounds, penicimarin (**63**), aspergillumarin A (**64**), aspergillumarin B (**65**), 5-hydroxysescandelin (**66**), sescandelin A (**67**), and sescandelin B (**68**), were isolated from the mangrove endophytic fungus *Hypoxylon* sp. Compounds **61 **and **65** exhibited DPPH radical scavenging activity, with IC_50_ values of 15.36 and 3.69 µM, respectively [27]. A new compound, 8-hydroxy-3-hydroxymethyl-6-methoxy-7-methylisocoumarin (**69**), was isolated from the mangrove endophytic fungus *Botryosphaeria ramosa*. Compound **69** exhibited inhibitory activity against *Fusarium oxysporum*, *Fusarium graminearum*, *Penicillium italicum*, and *Colletotrichum musae*, with IC_50_ values ranging from 52.97 to 847.46 µM. Its activity against some pathogens was stronger than that of the positive control triadimefon [28]. One compound, peniisocoumarin H (**70**), was isolated from the mangrove-derived fungus *Trichoderma harzianum* D13 [29]. A new compound, cytospomarin (**71**), was isolated from the mangrove-derived fungus *Cytospora* sp. Compound **71** exhibited weak inhibitory activity against *Escherichia coli* GIM1.201 and *Magnaporthe oryzae*, with MIC values of 0.35 and 1.41 mM, respectively [30].

Five new compounds, setosphamarins A-E (**72**–**76**), and three known compounds, 4,8-dihydroxy-3-((*R*)-2-hydroxypentyl)-6,7-dimethoxyisochroman-1-one (**77**), (3*R*,4*R*)-4,8-dihydroxy-3-(2-hydroxypentyl)-6,7-dimethoxyisochroman-1-one (**78**), and (3*R*,4*R*)-4,6,8-trihydroxy-3-((*R*)-2-hydroxypentyl)-7-methoxyisochroman-1-one (**79**), were isolated from the mangrove-derived fungus *Setosphaeria rostrate* [31]. Three new compounds, phomochromenones D-F (**80**–**82**), and two known compounds, diaporchromanone C (**83**) and diaporchromanone D **(84)**, were isolated from the mangrove sediment-derived fungus *Phomopsis asparagi* DHS-48 [32]. A new compound, incarxanthone E (**85**), was isolated from the mangrove endophytic fungus *Peniophora incarnate* Z4 [33]. Three new compounds, spiromastol M (**86**), (*P*, 9′*R*) spiromastol N (**87**), (*M*, 9′*R*) spiromastol N (**88**), and one known compound, palmaerin A (**89**), were isolated from the mangrove-derived fungus *Spiromastix* sp. SCSIO F190. Notably, compounds **87** and **88** were identified as a mixture of isomers. Compounds **86**–**88** exhibited significant antibacterial activity against methicillin-resistant *Staphylococcus aureus* (MRSA), *Enterococcus faecalis*, *Micrococcus luteus*, *Staphylococcus simulans*, *Enterococcus faecium* ATCC 29212, *Bacillus subtilis*, and *Enterococcus gallinarum* BS01. The MIC values for compound **86** ranging from 17.35 to 69.41 μM, while those for compounds **87**–**88** the MIC values ranging from 3.92 to 62.75 μM [34].

A new compound xylariachromanone A (**90**), was isolated from the mangrove endophytic fungus *Xylaria arbuscula* QYF [35]. Two compounds, 1-(8-hydroxy-1-oxoisochroman-3-yl)propyl 4′-(6′-hydroxy-8′-oxotetrahydrofuran-5′-yl)acetate (**91**) and 6′*α*-(3′-(1′-(8-hydroxy-1-oxoisochroman-3-yl)propoxy)-3′-oxoethyl)-8′-oxotetrahydro-furan-6′-yl butyrate (**92**), were isolated from the mangrove endophytic fungus *Bacillus amyloliquefaciens*. Compounds **91** and **92** demonstrated anti-inflammatory activity, as determined by a 5-LOX inhibition assay, with IC_50_ values of 1.23 and 1.11 mM, respectively [36]. A new compound asperisocoumarin G (**93**), was isolated from the mangrove endophytic fungus *Aspergillus* sp. 085242. Compound **93** exhibited α-glucosidase inhibitory activity with an IC_50_ value of 392.4 μM, which was superior to that of the positive control acarbose (IC_50_ = 725.1 μM) [37]. Two compounds, alternariol (**94**) and alternariol 4-methyl ether (**95**), were isolated from the mangrove endophytic fungus *Alternaria* sp. R6 [38]. One compound, alternariol (**96**), was isolated from the mangrove rhizosphere sediment-derived fungus *Arthrinium* sp. SCSIO 41305 [39].

### 2.2. Chromones

**Figure 2:**
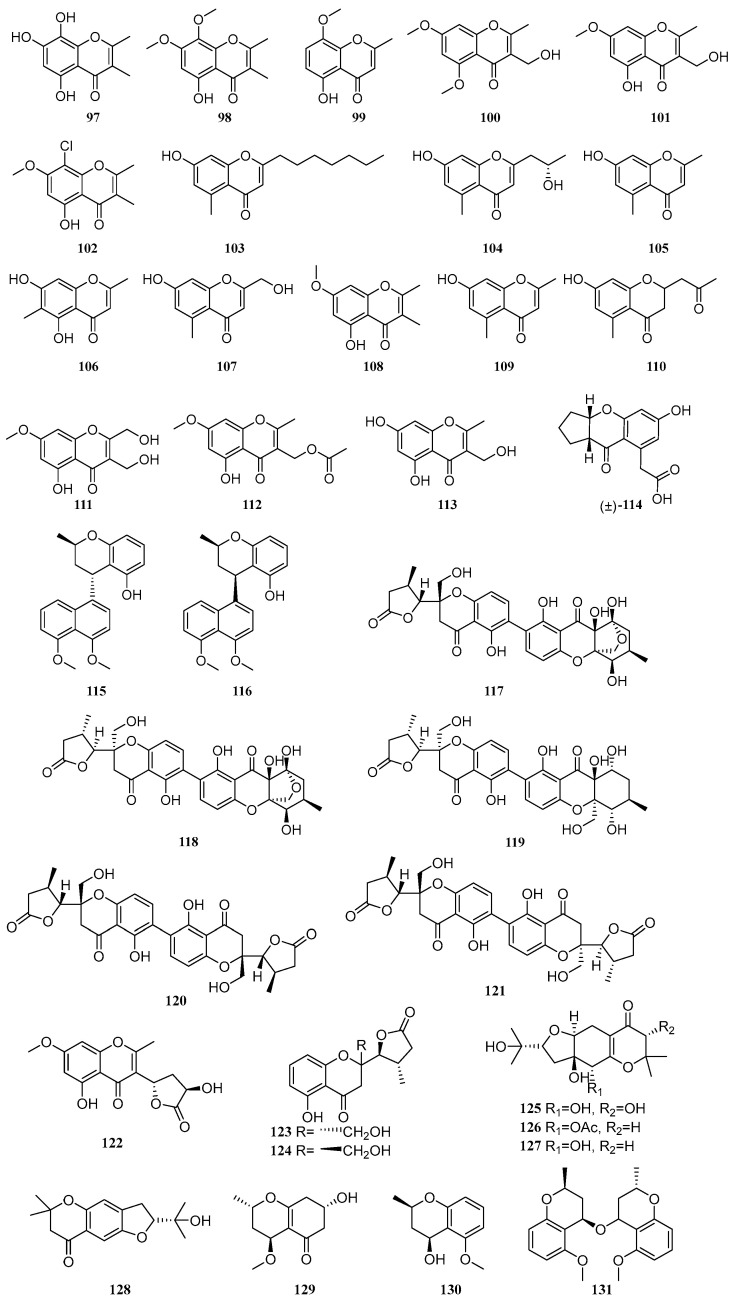
Chromones in polyketide compounds of mangrove fungi.

Chromones are a class of natural products with benzo-γ-pyrone as the core scaffold, which are widely distributed in plants and microorganisms. From 2020 to 2025, a total of 35 chromone compounds were isolated and identified from mangrove-derived fungi. Their structures are shown in (Figure 2), and 14 of these compounds exhibit biological activities such as antibacterial, antioxidant, and enzyme inhibitory effects.

Liu et al. employed the OSMAC strategy to isolate one new chromone, talamin E (**97**), and one known compound, talamin B (**98**), from the mangrove-derived fungus *Penicillium* sp. HDN15-312. Compound **97** exhibited good DPPH free radical scavenging activity with an IC_50_ value of 6.79 μM, which was more potent than that of the positive control, vitamin C [40]. One known compound, 5-hydroxy-8-methoxy-2-methyl-4*H*-1-benzopyran-4-one (**99**), were isolated from the mangrove endophytic fungus *Daldinia eschscholzii* MCZ-18 [9]. A new compound 3-(hydroxymethyl)-5,7-dimethoxy-2-methyl-4*H*-chromen-4-one (**100**), along with a known compound, 5-hydroxy-3-(hydroxymethyl)-7-methoxy-2-methyl-4*H*-chromen-4-one (**101**), were isolated from the mangrove-derived fungus *Trichoderma lentiforme* ML-P8-2. The IC_50_ values of compounds **100** and **101** against acetylcholinesterase (AChE) were 33.7 µM and 20.6 µM, respectively. Additionally, compound **101** exhibited moderate inhibitory activity against *Candida albicans*, with an MIC value of 25 µM [41]. A new compound 8-chloro-5-hydroxy-2,3-dimethyl-7-methoxychromone (**102**), was isolated from the mangrove-derived fungus *Mollisia* sp. SCSIO41409 [13]. A known compound, phomotone F (**103**), was isolated from the mangrove-derived fungus *Phomopsis* sp. QYM-13. Compound **103** demonstrated significant anti-inflammatory activity, with an IC_50_ value of 25.0 µM, which was stronger than that of the positive control L-NMMA (IC_50_ = 32.8 µM) [42]. A compound 2-(2′-hydroxypropyl)-5-methyl-7-hydroxychromone (**104**), was isolated from the co-culture fermentation products of two mangrove endophytic fungi, *Phomopsis asparagi* DHS-48 and *Phomopsis* sp. DHS-11. This compound showed weak inhibitory activity on ConA (T cell)- and LPS (B cell)-induced proliferation of mouse splenic lymphocytes, with IC_50_ values of 111.01 and 123.84 µM, respectively [43]. A known compound, 7-hydroxy-2,5-dimethylchromone (**105**), was isolated from the mangrove endophytic fungus *Epicoccum sorghinum*. Compound **105** significantly inhibited the growth of *Fusarium graminearum* and *Fusarium oxysporum*, both with an MIC value of 526.32 μM [44]. A known compound, eugenitol (**106**), was isolated from the mangrove endophytic fungus *Aspergillus* sp. SCSIO41407. Compound **106** exhibited weak inhibitory activity against methicillin-resistant *Staphylococcus aureus* (MRSA), with an MIC value of 485.4 µM [45].

A compound, 7-hydroxy-2-(hydroxymethyl)-5-methyl-4*H*-chromen-4-one (**107**), was isolated from the mangrove-derived fungus *Penicillium janthinellum* [46]. A compound 5-hydroxy-2,3-dimethyl-7-methoxychromone (**108**), was isolated from the mangrove sediment-derived fungus *Trichoderma harzianum* SCSIO 41051 [22]. Hu et al. isolated two compounds, altechromone A (**109**) and aloesone (**110**), from the mangrove soil-derived fungus *Arthrinium* sp. SCSIO 41305 [39]. In another study, Hu et al. identified three new compounds, 5-hydroxy-2,3-dihydroxymethyl-7-methoxychromone (**111**), 5-hydroxy-3-acetoxymethyl-2-methyl-7-methoxychromone (**112**), and 5,7-dihydroxy-3-hydroxymethyl-2-methylchromone (**113**), from the mangrove endophytic fungus *Botryosphaeria ramose*. Compounds **111**–**113** exhibited antimicrobial activities against *Fusarium oxysporum*, *Fusarium graminearum*, *Penicillium italicum*, and *Colletotrichum musae*, with IC_50_ values ranging from 24.8 to 793.65 µM. Some of the compounds showed stronger activity than the positive control triadimefon [28]. A new compound, curvulanone (**114**), featuring a rare 3-acetylchromone scaffold, was isolated from the mangrove endophytic fungus *Curvularia aeria*. The structure of **114** was unequivocally determined by X-ray single-crystal diffraction. Biological evaluation revealed that compound **114** inhibited monoamine oxidase B (MAO-B) with an IC_50_ of 55.8 µM, while exhibiting weaker inhibition against MAO-A (IC_50_ = 117.9 µM) and sirtuin 1 (SIRT1, IC_50_ = 107.9 µM). A putative biosynthetic pathway for **114** was also proposed [47]. Two new compounds, cladonaphchroms A (**115**) and B (**116**), were isolated from the mangrove endophytic fungus *Cladosporium* sp. JJM22. Compound **115** displayed significant antibacterial activity against *Staphylococcus albus* ATCC 8799 with an MIC of 3.57 μM, and also inhibited *Escherichia coli* ATCC 25922, *Bacillus subtilis* ATCC 6633, *Micrococcus tetragenus* ATCC 13623, and *Micrococcus luteus* ATCC 9341, with MIC values ranging from 7.14 to 28.57 μM. Additionally, compounds **115** and **116** showed antifungal activities against *Alternaria brassicicola*, *Phytophthora parasitica* var. *nicotianae*, *Colletotrichum capsici*, *Bipolaris oryzae*, *Diaporthe medusaea*, and *Ceratocystis paradoxa*, with MIC values between 71.43 and 285.71 μM [48].

Guided by metabolomics, three new compounds, phomoxanthones L-N (**117**–**119**), along with two known compounds, phomopsis-H76A (**120**) and diaporthochromone B (**121**), were isolated from the co-culture fermentation products of two mangrove endophytic fungi, *Phomopsis asparagi* DHS-48 and *Phomopsis* sp. DHS-11 [49]. A new compound 5-hydroxy-3-((3′*R*,5′*S*)-3′-hydroxy-2′-oxotetrahydrofuran-5′-yl)-7-methoxy-2-methyl-4*H*-chromen-4-one (**122**), was isolated from the mangrove-derived fungus *Trichoderma lentiforme* ML-P8-2. Compound **122** exhibited moderate inhibitory activity against acetylcholinesterase (AChE) with an IC_50_ value of 38.6 µM, as well as moderate anti-fungal activity against *Candida albicans*, showing an MIC value of 50 µM [41]. Two known compounds, mycochromone A (**123**) and mycochromone B (**124**), were isolated from the mangrove endophytic fungus *Mycosphaerella* sp. L3A1. The absolute configurations of compounds **123** and **124** were determined using X-ray single-crystal diffraction with CuKα radiation and electronic circular dichroism (ECD) calculations [50]. Two new compounds, pestalotheols P-Q (**125**–**126**), and two known compounds, pestalotheol A (**127**) and pestalotheol D (**128**), were isolated from the mangrove endophytic fungus *Pseudopestalotiopsis theae* [51]. A new chromone derivative, xylariaone A (**129**), was isolated from the mangrove endophytic fungus *Xylaria arbuscula* QYF. Its absolute configuration was established via Mosher’s ester method [35]. Two new compounds, (2*R*,4*S*)-5-methoxy-2-methyl-2*H*-1-benzopyran-4-ol (**130**) and (2*S*,2′*S*,4*R*,4′*R*)-bis(5-methoxy-2-methyl-2*H*-1-benzopyran)-4-ether (**131**), were isolated from the mangrove endophytic fungus *Penicillium citrinum* QJF-22 [52].

### 2.3. Xanthones

**Figure 3:**
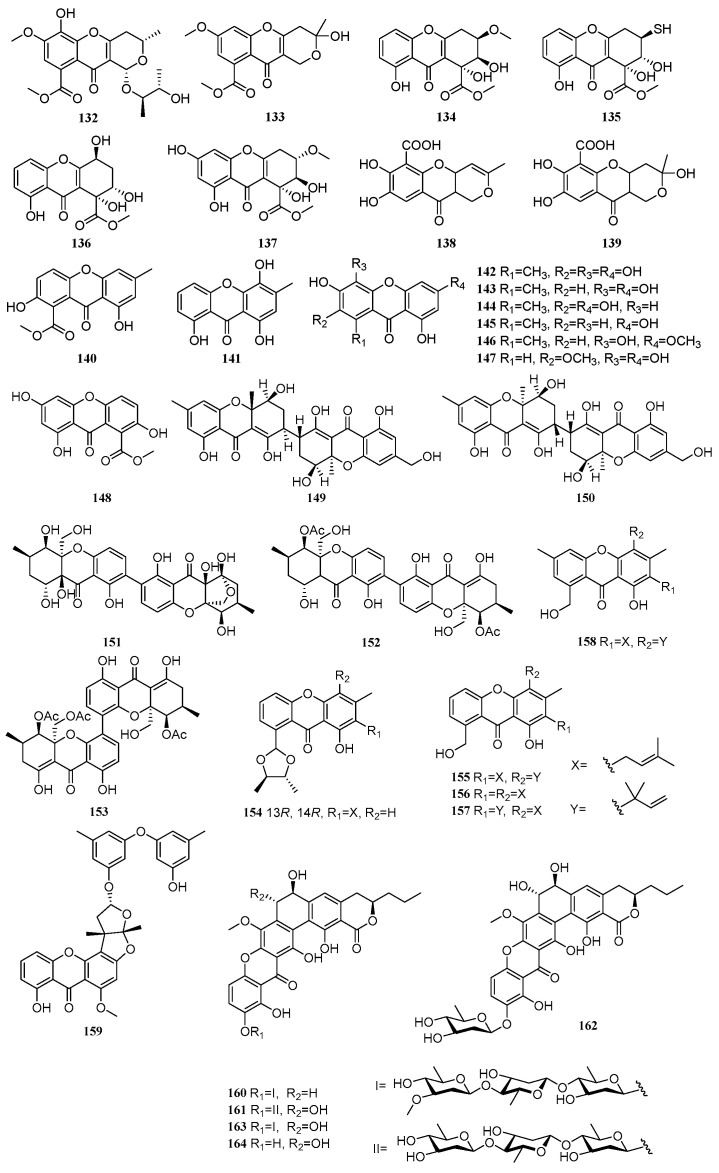
Xanthones in polyketide compounds of mangrove fungi.

Between 2020 and 2025, a total of 33 xanthone derivatives were isolated and characterized from mangrove-derived fungi. Their structures are shown in Figure 3. Among these, 25 compounds exhibited various biological activities, including antitumor, anti-inflammatory, and antimicrobial effects.

A new compound, phomochromenone G (**132**), and one known compound, diaporchromone A (**133**), were isolated from the mangrove sediment-derived fungus *Phomopsis asparagi* DHS-48. Compound **133** exhibited moderate to weak immunosuppressive activity against T and B lymphocytes, with IC_50_ values of 34 and 117 µM, respectively [32]. Three new compounds, incarxanthones A–C (**134**–**136**), and one known compound, globosuxanthone B (**137**), were isolated from the mangrove endophytic fungus *Peniophora incarnate* Z4. Compound **135** showed inhibitory activity against three tumor cell lines: human melanoma cells (A375), human breast cancer cells (MCF-7), and human leukemia cells (HL-60), with IC_50_ values of 8.6, 6.5, and 4.9 µM, respectively [33]. Two known compounds, penialidin C (**138**) and penialidin A (**139**), were isolated from the mangrove-derived fungus *Penicillium javanicum*. Compounds **138** and **139** exhibited moderate to strong inhibitory activities against four strains of *Staphylococcus aureus*. Notably, compound **138** showed significant antibacterial activity against methicillin-resistant *S. aureus* (MRSA) ATCC 43300, with an MIC value of 2.67 µM, comparable to the positive control vancomycin (0.54 µM). It was also active against three other MRSA strains (ATCC 33591, ATCC 25923, and ATCC 29213), with MIC values ranging from 21.40 to 85.62 µM. Compound **139** exhibited antibacterial activity against MRSA ATCC 43300 and *S. aureus* ATCC 29213, with MIC values of 10.10 and 40.32 µM, respectively. At a concentration of 50 µg/mL, compound **139** also inhibited the growth of *Alternaria alternata*, with an inhibition rate of 56.8% [53]. One compound, pinselin (**140**), were isolated from the mangrove sediment-derived fungus *Talaromyces* sp. SCSIO 41428 [23]. A known compound, ravenelin (**141**), was isolated from the mangrove-derived fungus *Setosphaeria rostrata*. Its anti-inflammatory activity was evaluated by measuring NO production in LPS-induced J774A.1 macrophage cells. Compound **141** demonstrated significant inhibitory activity with an IC_50_ value of 6.27 µM. Mechanistic studies revealed that it suppressed the expression of iNOS and COX-2 [31]. Six compounds, anomalin B (**142**), 1,3,5,6-tetrahydroxy-8-methylxanthone (**143**), anomalin A (**144**), 1,3,6-trihydroxy-8-methylxanthone (**145**), 3,4,8-trihydroxy-6-methoxy-1-methylxanthone (**146**), and caloxanthone E (**147**), were isolated from the mangrove soil-derived fungus *Arthrinium* sp. SCSIO 41305. Compounds **142**, **143**, **145**, and **147** showed moderate inhibitory activity against neuraminidase (NA), with inhibition rates of 83.30%, 91.46%, 75.72%, and 77.46% at 100 µg/mL, respectively. Further testing indicated that only compound **143** exhibited weak inhibition against AChE, with an inhibition rate of 52.14% at 50 µg. Compounds **143**–**147** showed weak enzyme inhibitory activity against phosphatidylinositol 3-kinase (PI3K), with IC_50_ values of 1.07, 4.41, 1.93, 2.90, and 3.32 µM, respectively [39]. A new compound, 2,8-dihydroxyvertixanthone (**148**), was isolated from the mangrove endophytic fungus *Peniophora incarnate* Z4 [33]. Two new compounds, aflaxanthones A (**149**) and B (**150**), were isolated from the mangrove endophytic fungus *Aspergillus* flavus QQYZ. Compound **149** exhibited good inhibitory activity against *Colletotrichum gloeosporioides* with an MIC of 3.13 µM (positive control ketoconazole, MIC = 0.1 µM), and moderate activity against *Fusarium oxysporum* and *Candida albicans* (MIC = 12.5 µM). Compound **150** showed moderate activity against *F. oxysporum* and *Colletotrichum musae* (MIC = 12.5 µM). Compound **149** also displayed moderate activity against MRSA (MIC = 12.5 µM) and inhibited *Bacillus subtilis* ATCC 6633 (MIC = 25 µM, positive control ampicillin, MIC = 0.39 µM) [54]. Three compounds, phomoxanthone D (**151**), dicerandrol (**152**), and 12-*O*-deacetyl-phomoxanthone A (**153**), were isolated from the co-culture fermentation products of two mangrove endophytic fungi, *Phomopsis asparagi* DHS-48 and *Phomopsis* sp. DHS-11. Compounds **152** and **153** exhibited significant cytotoxicity against human liver cancer cells (HepG-2), with IC_50_ values ranging from 4.83 to 12.06 µM. Compound **151** showed weak immunosuppressive activity on ConA-induced (T cell) and LPS-induced (B cell) proliferation of mouse splenic lymphocytes [49]. Five new compounds, staprexanthones A-E (**154**–**158**), were isolated from the mangrove endophytic fungus *Stachybotrys chartarum*. Compounds **154**, **155**, and **158** significantly increased β-cell numbers in zebrafish. Compounds **155** and **158** enhanced β-cell mass by promoting cell cycle progression at the G1/S transition, suggesting their potential as novel anti-diabetic agents through stimulation of β-cell regeneration [55]. A new compound, rhizoaspergillinol A (**159**), was isolated from the mangrove endophytic fungus *Aspergillus* sp. A1E3. Compound **159** exhibited potent anti-proliferative activity against three tumor cell lines, HepG2, LLC, and B16-F10, with IC_50_ values of 8.83, 14.18, and 15.12 µM, respectively. It induced G2/M phase arrest in HepG2 cells in a dose-dependent manner [56]. Three new compounds, kebanmycins A-C (**160**–**162**), and two known compounds, FD-594 (**163**) and its aglycon (**164**), were isolated from the mangrove-derived fungus *Streptomyces* sp. SCSIO 40068. Compounds **160**–**164** were active against a panel of Gram-positive bacteria, including *S. aureus* ATCC 29213, MRSA shhs-A1, MRSA 1862, MRSA 669, MRSA 991, *B. subtilis* 1064, *V. alginolyticus* ATCC 13214, and Gram-negative bacteria, *A. baumannii* 19606. Compound **160** showed remarkable antibacterial activity, particularly against *S. aureus* ATCC 29213, MRSA shhs-A1, and MRSA 1862, with a uniform MIC of 0.125 µg/mL. It also exhibited more potent antitumor activity than compound **163**, significantly inhibiting HepG2 and MCF-7 cells with IC_50_ values of 0.25 µM and 0.12 µM, respectively, outperforming the positive control doxorubicin (IC_50_ = 3.1 and 0.72 µM). This finding highlights the importance of the absence of the 7-OH group for enhancing antibacterial activity. Through in vitro biochemical characterization, the involvement of the methyltransferase KebMT2 was demonstrated, and a biosynthetic pathway for the compounds was proposed [57].

### 2.4. Quinones

**Figure 4:**
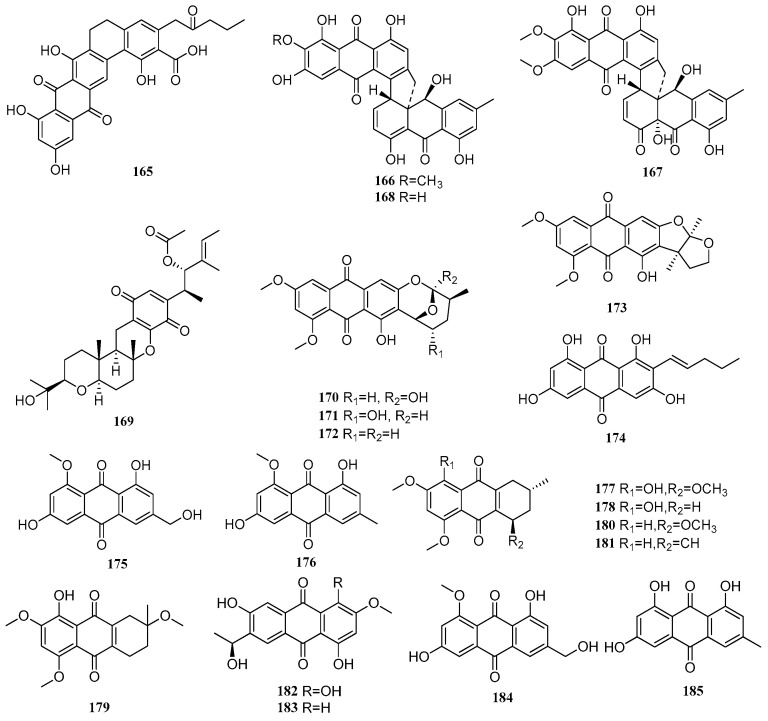

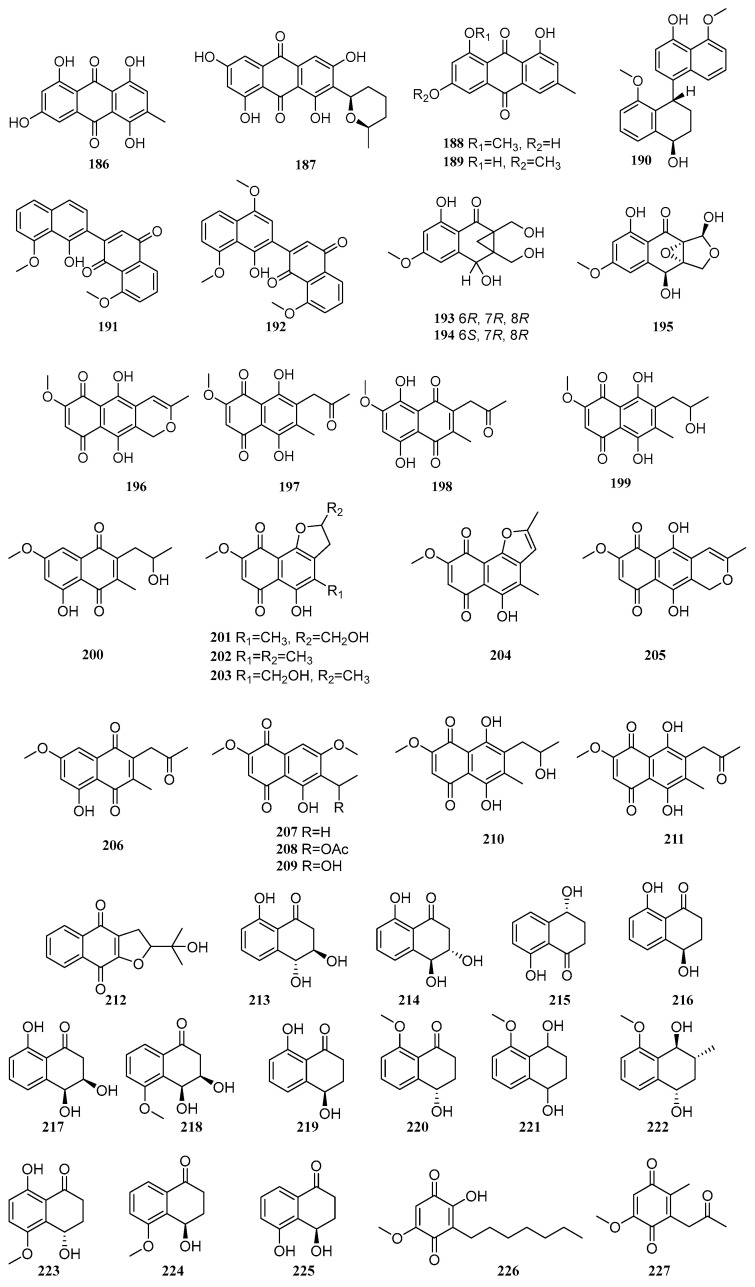
Quinones in polyketide compounds of mangrove fungi.

Between 2020 and 2025, a total of 63 quinone compounds were isolated and identified from mangrove-derived fungi. Their structures are shown in Figure 4. Among these, 37 compounds exhibited various biological activities, including anti-inflammatory, antitumor, and antimicrobial effects.

A new compound, kebanmycin D (**165**), was isolated from the mangrove-derived fungus *Streptomyces* sp. SCSIO 40068. Compound **165** showed antibacterial activity against a range of Gram-positive bacteria, including *S. aureus* ATCC 29213, MRSA shhs-A1, MRSA 1862, MRSA 669, and MRSA 991, with MIC values ranging from 31.87 to 63.75 μM [57]. Two new compounds, parengyomarin A (**166**) and parengyomarin B (**167**), along with one known compound, torrubiellin B (**168**), were isolated from the mangrove endophytic fungus *Parengyodontium album*. Compounds **166**–**168** exhibited significant antibacterial activity against both *Staphylococcus aureus* and methicillin-resistant *S. aureus* (MRSA), with MIC values between 0.39 and 3.12 μM [58]. A known compound, stemphone C (**169**), was isolated from the mangrove-derived fungus *Mollisia* sp. SCSIO 41409. The absolute configuration of **169** was determined for the first time via X-ray single-crystal diffraction analysis. The compound displayed varying degrees of antibacterial activity against *Erysipelothrix rhusiopathiae* WH13013 and *Streptococcus suis* SC19, with IC_50_ values of 3.04 and 12.16 µM, respectively, comparable to the positive control penicillin (MIC = 19.53 µM). In addition, compound **169** exhibited broad-spectrum cytotoxicity against seven tumor cell lines (22Rv1, PC-3, HepG2, A549, HeLa, WPMY-1, and MC3T3-E1), with IC_50_ values ranging from 2.11 to 11.68 µM. It showed particularly potent anti-proliferative activity against the human prostate cancer cell line PC-3 (IC_50_ = 2.77 µM). Further studies revealed that **169** exerted its anti-proliferative effects by reducing colony formation, inducing apoptosis, and arresting the cell cycle in PC-3 cells [13].

One new compound, asperquinone A (**170**), and four known compounds, 6,8-di-*O*-methylnidurufin (**171**), 6,8-di-*O*-methylaverufin (**172**), aversin (**173**), and averythrin (**174**), were isolated from the mangrove endophytic fungus *Aspergillus* sp. 16-5C. These compounds (**170**–**174**) were preliminarily screened for inhibitory activity against *Mycobacterium tuberculosis* protein tyrosine phosphatase B (MptpB), but none showed significant inhibition (IC_50_ > 60 µg/mL) [59]. Two known compounds, questinol (**175**) and questin (**176**), were isolated from the mangrove endophytic fungus *Aspergillus* sp. SCSIO 41407 [45]. Two new compounds, 6-hydroxy-astropaquinone B (**177**) and astropaquinone D (**178**), and three known compounds, 3-*O*-methyl-9-*O*-methylfusarubin (**179**), (1*R*,3*S*)-6-hydroxy-astropaquinone B (**180**), and (1*R*,3*S*)-6-hydroxy-astropaquinone C (**181**), from the mangrove endophytic fungus *Fusarium napiforme*. Compounds **177**–**179** exhibited antibacterial activity against *Staphylococcus aureus*, with MIC values of 18.98, 41.39, and 18.98 μM, respectively. They also showed moderate antibacterial effects against *Pseudomonas aeruginosa*, all with MIC values ranging from 18.98 to 20.86 μM [60].

Two new compounds, (11*S*)-1,4,6-trihydroxy-7-(1-hydroxyethyl)-3-methoxyanthracene-9,10-dione (**182**) and (11*S*)-1,6-dihydroxy-7-(1-hydroxyethyl)-3-methoxyanthracene-9,10-dione (**183**), were isolated from the mangrove endophytic fungus *Fusarium* sp. J3-2. Compound **182** exhibited weak to moderate antibacterial activity against five pathogenic strains, *Staphylococcus aureus* ATCC 43300, ATCC 25923, ATCC 29213, *Enterococcus faecalis* ATCC 51299, and *Enterococcus faecium* ATCC 35667, with MIC values ranging from 75.76 to 151.52 μM. In addition, both compounds **182** and **183** demonstrated anti-fouling activity, completely inhibiting the attachment of barnacle larvae (attachment rate = 0%) [61]. Three known compounds, questinol (**184**), emodin (**185**), and catenarin (**186**), were isolated from the mangrove endophytic fungus *Aspergillus* sp. WHUF0343. Compounds **184** and **185** exhibited antibacterial activity against *Staphylococcus aureus* ATCC 25923 and methicillin-resistant *Staphylococcus aureus* NRS271, with MIC values between 29.63 and 59.26 µM. Compound **186** also showed strong inhibitory activity against four strains of *Helicobacter pylori* (26695, G27, 159, and 129), with MIC values ranging from 3.50 to 13.99 µM [62]. A known compound, averufanin (**187**), was isolated from the mangrove endophytic fungus *Aspergillus* sp. A1E3. The absolute configuration of **187** was determined for the first time via ECD calculations [56]. Two known compounds, questin (**188**) and physcion (**189**), were isolated from the mangrove endophytic fungus *Aspergillus fumigatus* HQD24. Compound **188** exhibited inhibitory activity on LPS-induced B-cell proliferation (IC_50_ = 108.67 μM) and ConA-induced T-cell proliferation (IC_50_ = 41.67 μM) [19]. In another study, Xu et al. isolated two new compounds, dalesconosides C-D (**190**–**191**), and one new natural product, dalesconoside E (**192**), from the mangrove endophytic fungus *Daldinia eschscholzii* MCZ-18. Compound **190** displayed broad-spectrum antibacterial activity against five pathogenic microorganisms, *Enterococcus faecalis*, methicillin-resistant *Staphylococcus aureus*, *Escherichia coli*, *Pseudomonas aeruginosa*, and *Candida albicans*, with IC_50_ values ranging from 12.5 to 50 μM [9].

One new compound (6*R*,7*R*,8*R*)-theissenone A (**193**), and two known compounds, (6*S*,7*R*,8*R*)-theissenone (**194**) and arthrinone (**195**), were isolated from the mangrove endophytic fungus *Arthrinium marii* M-211. The IC_50_ values of compounds **193**–**195** against rat hepatoma H4IIE cells were 67.5, 46.6, and 13.4 μM, respectively (positive control staurosporine: IC_50_ = 20.9 nM). Compounds **193** and **194** showed moderate antibacterial activity against both *Pseudomonas aeruginosa* ATCC 15442 and *Staphylococcus aureus* NBRC 13276, with a uniform MIC of 25 μM, while compound **195** exhibited moderate antibacterial activity against the same strains with an MIC of 12.5 μM [63]. Four known compounds, anhydrofusarubin (**196**), javanicin (**197**), dihydrojavanicin (**198**), and solaniol (**199**), were isolated from the mangrove-derived fungus *Lasiodiplodia theobromae*. Compounds **197**–**199** displayed notable inhibitory activity against *Trypanosoma brucei*, with MIC values of 0.60, 0.32, and 1.90 μM, respectively [18].

Two new compounds, talanaphthoquinones A-B (**200**–**201**), along with ten known compounds, anhydrojavanicin (**202**), 2,3-dihydro-5-hydroxy-4-hydroxymethyl-8-methoxy-2-methylnaphtho[1,2-b]furan-6,9-dione (**203**), anhydrojavanicin (**204**), anhydrofusarubin (**205**), 2-acetonyl-3-methyl-5-hydroxy-7-methoxynaphthazarin (**206**), 6-ethyl-2,7-dimethoxyjuglone (**207**), 6-[1-(acetyloxy)ethyl]-5-hydroxy-2,7-dimethoxy-1,4-naphthalenedione (**208**), 5-hydroxy-6-(1-hydroxyethyl)-2,7-dimethoxy-1,4-naphthalenedione (**209**), solaniol (**210**), and javanicin (**211**), were isolated from the mangrove endophytic fungus *Talaromyces* sp. SK-S009. All compounds except **201** inhibited NO production induced by LPS, with IC_50_ values ranging from 3.9 to 22.6 µM, which were lower than that of the positive control indomethacin (26.3 µM). Compound **208** suppressed the mRNA expression of inducible nitric oxide synthase (iNOS) and cyclooxygenase-2 (COX-2) in RAW264.7 macrophages. Furthermore, it reduced the mRNA levels of pro-inflammatory cytokines interleukin (IL-1β, IL-6) and tumor necrosis factor (TNF-α) [64]. A known compound, stenocarpoquinone B (**212**), were isolated from the mangrove endophytic fungus *Avicennia officinalis* [65]. A known compound, *trans*-3,4-dihydro-3,4,8-trihydroxynaphthalen-1(2*H*)-one (**213**), was isolated from the mangrove endophytic fungus *Penicillium polonicum* H175 [66]. A known compound, *trans*-3,4-dihydro-3,4,8-trihydroxynaphthalen-1(2*H*)-one (**214**), was isolated from the mangrove sediment-derived fungus *Roussoella* sp. SCSIO 41427 [10]. A known compound, (4*S*)-4,8-dihydroxy-α-tetralone (**215**), was isolated from the mangrove-derived fungus *Colletotrichum* sp. J065 [67]. A known compound, regiolone (**216**), was isolated from the mangrove-derived fungus *Cytospora* sp. Compound **216** exhibited weak antibacterial activity against *Bacillus subtilis*, *Colletotrichum gloeosporioides*, and *Magnaporthe oryzae*, with a uniform IC_50_ value of 561.6 µM [68]. A known compound, *cis*-(3*R*,4*S*)-3,4-dihydro-3,4,8-trihydroxynaphthalen-1(2*H*)-one (**217**), was isolated from the mangrove endophytic fungus *Penicillium citrinum* QJF-22. Compound **217** exhibited moderate anti-inflammatory activity by inhibiting LPS-induced NO release in RAW264.7 cells, with an IC_50_ value of 44.7 µM, and showed no cytotoxicity toward RAW264.7 cells at concentrations up to 50 µM [52].

A new compound dalesconoside F (**218**), and seven known compounds, regiolone (**219**), nodulisporone (**220**), nodulisporol (**221**), xylariol A (**222**), (4*R*)-4,8-dihydroxy-3-hydro-5-methoxy-1-naphthalenone (**223**), (4*R*)-*O*-methylsclerone (**224**), and (4*R*)-3,4-dihydro-4,5-dihydroxynaphthalen-1(2*H*)-one (**225**), were isolated from the mangrove endophytic fungus *Daldinia eschscholzii* MCZ-18. Compounds **223**–**225** exhibited antibacterial activity against five pathogenic bacteria, with IC_50_ values ranging from 6.25 to 50 µM [9]. A new natural product, embelin A (**226**), was isolated from the mangrove-derived fungus *Penicillium* sp. SCSIO 41411. Its absolute configuration was determined for the first time via X-ray single-crystal diffraction. Compound **226** displayed cytotoxic activity against prostate cancer cell lines PC-3 and LNCaP, with IC_50_ values of 18.69 and 31.62 µM, respectively [8]. A known compound anserinone A (**227**), was isolated from the mangrove-derived fungus TBRC-BCC 64093 [12].

### 2.5. Lactones

**Figure 5:**
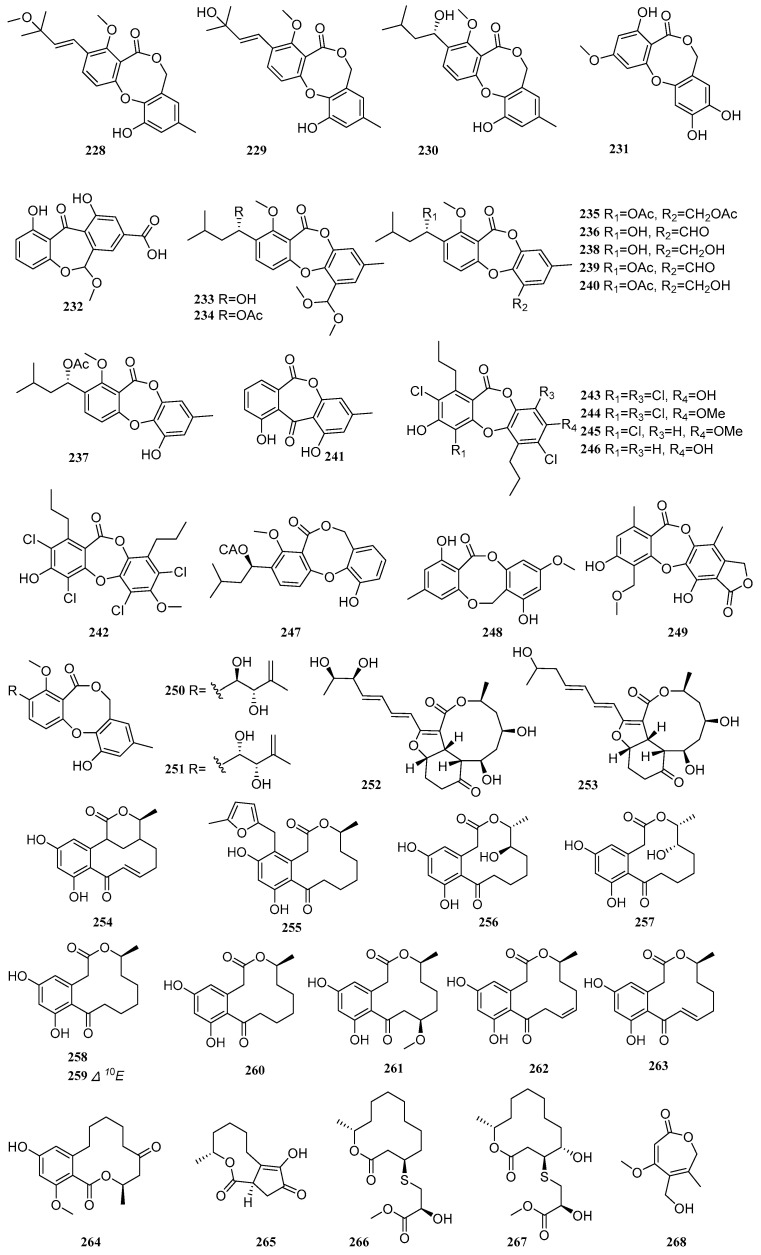

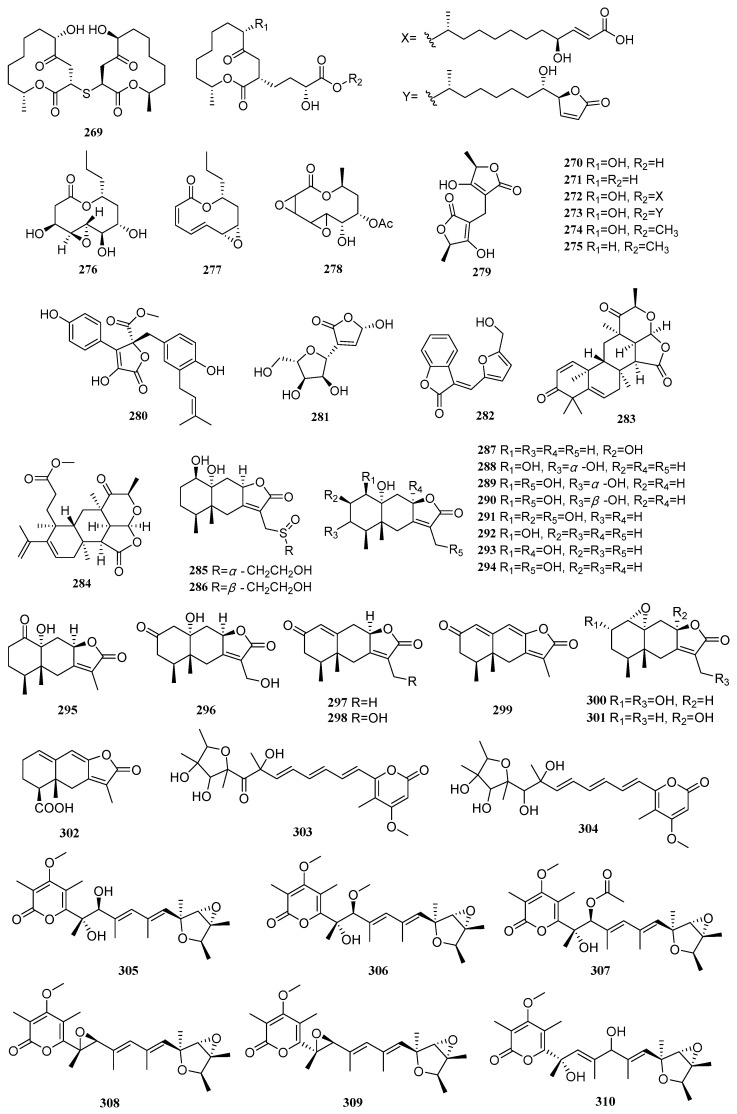

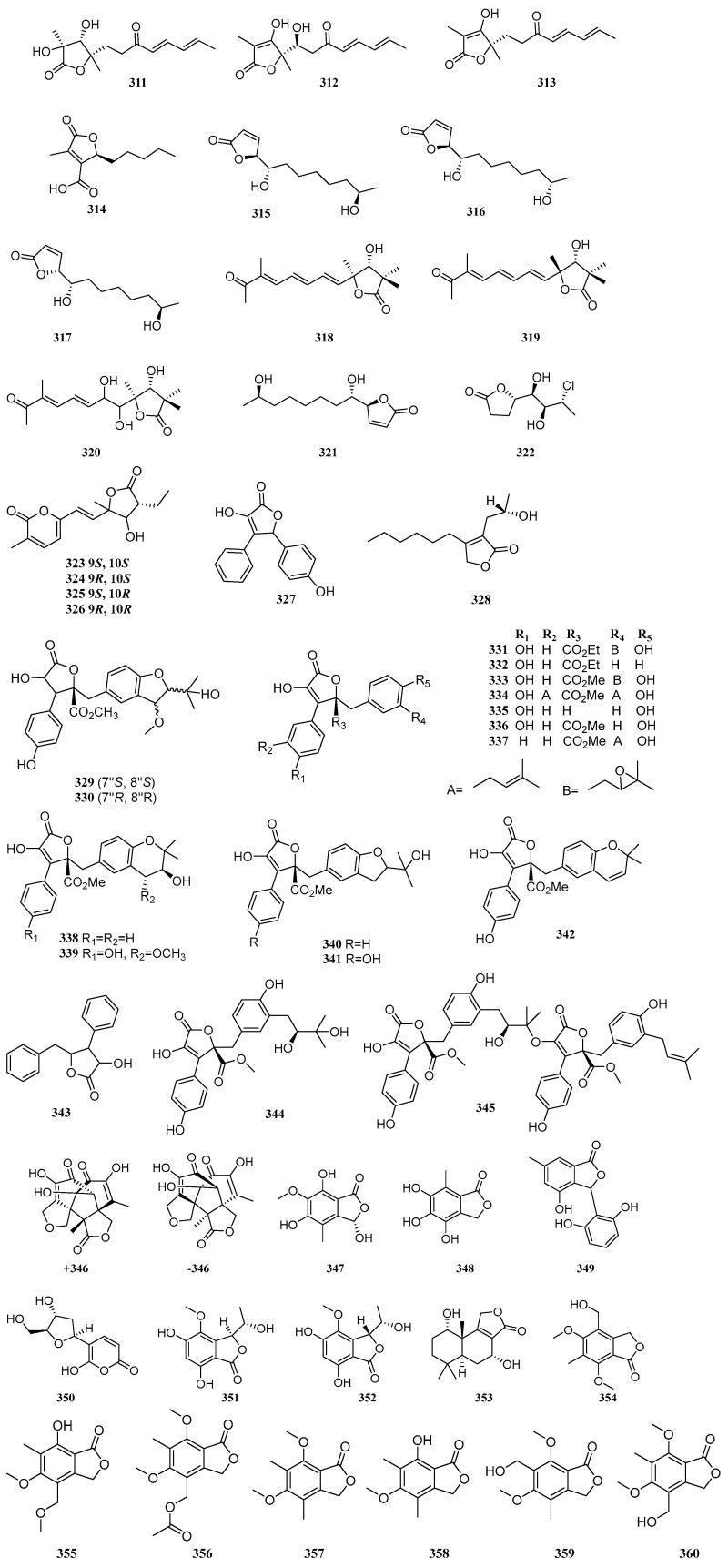

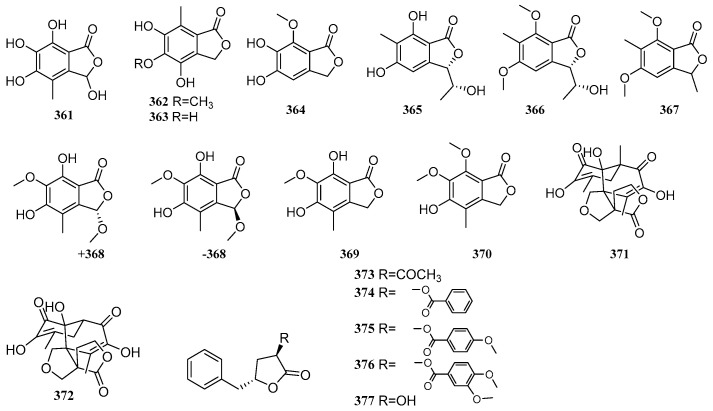
Lactones in polyketide compounds of mangrove fungi.

Lactones represent a major class of secondary metabolites from mangrove-derived fungi. These cyclic organic molecules, composed of carboxylate esters, are formed through the dehydration of lactic acid. Based on ring size, they can be categorized into macrolides, sesquiterpene lactones, among others. Macrolides often exhibit antibacterial properties, while sesquiterpene lactones are noted for their antimalarial and immunomodulatory activities. Between 2020 and 2025, a total of 150 lactone compounds were isolated and identified from mangrove-derived fungi. Their structures are shown in Figure 5.

Four compounds, alterlactone (**228**), penicillide (**229**), dehydroisopenicillide (**230**), and 3′-*O*-methyldehydroisopenicillide (**231**), were isolated from the mangrove-derived fungus *Talaromyces* sp. Compounds **229**–**231** exhibited antibacterial activity against *Staphylococcus aureus*, with MIC values of 50, 50, and 25 µg/mL, respectively. Compound **228** showed DPPH free radical scavenging activity with an EC_50_ value of 96.51 µM, which was weaker than that of the positive control vitamin C (EC_50_ = 72.39 µM) [69]. Liu et al., employing an OSMAC strategy, identified a new compound arugosinacid A (**232**), from the mangrove-derived fungus *Penicillium* sp. HDN15-312. Compound **232** exhibited moderate DPPH radical scavenging activity, with an IC_50_ value of 56.92 μM [40]. Four new compounds, talaronins A-D (**233**–**236**), and five known compounds, purpactin A (**237**), talaromyone A (**238**), purpactin C (**239**), talaromyone B (**240**), and alternaphenol B (**241**), were isolated from the mangrove-derived fungus *Talaromyces* sp. WHUF0362. Compounds **237** and **238** showed potent activity against four strains of *Helicobacter pylori* (26695, G27, 159, and 129), with MIC values ranging from 2.42 to 36.04 μM [11]. Five known compounds, spiromastixones L (**242**), I (**243**), J (**244**), G (**245**), and E (**246**), were isolated from the mangrove-derived fungus *Spiromastix* sp. SCSIO F190. Compounds **242-246** exhibited significant antibacterial activity against methicillin-resistant *Staphylococcus aureus* (MRSA), *Enterococcus faecalis*, *Micrococcus luteus*, *Staphylococcus simulans*, *Enterococcus faecium* ATCC 29212, *Bacillus subtilis*, and *Enterococcus gallinarum* BS01, with MIC values ranging from 0.125 to 32 μg/mL. Compound **244** was particularly potent, with MIC values between 0.125 and 4 μg/mL. Structure-activity relationship studies indicated that the presence of both ester and ether bonds linking rings A and B in compound **244** was crucial for its high activity, suggesting that the absence of an ether bond leads to a marked reduction in antibacterial efficacy [34]. A known compound, purpactin A (**247**), were isolated from the mangrove endophytic fungus *Penicillium* sp. TGM112. The compound exhibited moderate antioxidant activity, with an IC_50_ value of 4.6 mM [24]. A known compound, barceloneic lactone (**248**), was isolated from the mangrove endophytic fungus *Epicoccum sorghinum* [44]. A new compound, guanxidone A (**249**), was isolated from the mangrove endophytic fungus *Aspergillus* sp. GXNU-A9. It significantly reduced NO production in LPS-induced RAW264.7 cells, with an IC_50_ value of 8.22 μM [15].

Two known compounds, pestalotiollides A-B (**250**–**251**), were isolated from the mangrove-derived fungus *Pestalotiopsis* sp. HHL101 [20]. Two new compounds, colletotrikalactones A and B (**252**–**253**), from the mangrove-derived fungus *Colletotrichum* sp. J065 [67]. A known compound, α,β-dehydrocurvularin (**254**), was isolated from the mangrove endophytic fungus *Trichoderma* sp. FM652. It significantly inhibited the TNF-α-induced NF-κB pathway with an IC_50_ value of 14.63 µM. Compound **254** also exhibited moderate antibacterial activity against *Staphylococcus aureus* ATCC 12600 and methicillin-resistant *Staphylococcus aureus* ATCC 43300, with an MIC value of 33.11 µM, and inhibited *Bacillus subtilis* ATCC 6633 with an MIC value of 66.22 µM [70]. Three new compounds, sumalarins D, F-G (**255**–**257**), and two known compounds, curvularin (**258**) and dehydrocurvularin (**259**), were isolated from the mangrove-derived fungus *Penicillium sumatrense* MA-325. Compounds **255** and **258**–**259** exhibited inhibitory activity against the aquatic pathogens *Vibrio alginolyticus* and *Vibrio harveyi*, with MIC values ranging from 13.70 to 219.18 µM. Furthermore, compound **259** showed cytotoxic activity against tumor cell lines 5673, HCT 116, 786-O, and HeLa, with IC_50_ values of 3.5, 10.6, 10.9, and 14.9 µM, respectively [71]. Four known compounds, curvularin (**260**), 11-β-methoxycurvularin (**261**), β,γ-dehydrocurvularin (**262**), and α,β-dehydrocurvularin (**263**), were isolated from the mangrove endophytic fungus *Alternaria longipes*, and proposed a plausible biosynthetic pathway for compounds **260**–**263** [72]. A known compound, 6-oxolasiodiplodin (**264**), was isolated from the mangrove endophytic fungus *Trichoderma erinaceum* F1-1 [73].

Three new compounds cladocladosin A (**265**) and thiocladospolides F-G (**266**–**267**), were isolated from the mangrove endophytic fungus *Cladosporium cladosporioides* MA-299. Compound **265** features a novel carbon skeleton with a 5/9 bicyclic ring system, and a biosynthetic pathway for compounds **265**–**267** was proposed. Compounds **265**–**267** showed activity against the aquatic pathogens *Edwardsiella tarda* and *Vibrio anguillarum*, with MIC values ranging from 4.46 to 11.49 µM. Compound **265** was active against *Pseudomonas aeruginosa*, and compound **266** showed activity against the plant pathogenic fungus *Helminthosporium maydis*, both with MIC values of 17.86 and12.05 µM, respectively [74]. A new compound, botroxepinone (**268**), was isolated from the mangrove endophytic fungus *Botryosphaeria ramose*. It exhibited antimicrobial activity against *Fusarium oxysporum*, *Fusarium graminearum*, and *Colletotrichum musae*, with IC_50_ values ranging from 25 to 200 µg/mL, some of which were stronger than the positive control triadimefon [28]. Five new compounds, thiocladospolides F-J (**269**–**273**), and two known compounds, pandangolide (**274**) and thiocladospolide A (**275**), were isolated from the mangrove endophytic fungus *Cladosporium oxysporum*. Compound **270** exhibited broad-spectrum antibacterial activity against multiple pathogens, including *Cytospora mandshurica* Miura, *Colletotrichum gloeosporioides*, *Fusarium oxysporum* f. sp. *cucumerinum*, *Edwardsiella tarda*, and *Edwardsiella ictaluri*, with MIC values ranging from 4 to 32 µg/mL [75]. One new compound, asperlactone A (**276**), and two known compounds, (6*Z*,8*E*)-3-propyl-4,11-dioxa-bicyclo[8.1.0]undeca-6,8-dien-5-one (**277**) and 8-*O*-acetyl-5,6-dihydro-5,6-epoxymultiplolide A (**278**), were isolated from the mangrove endophytic fungus *Aspergillus* sp. GXNU-A9. Compounds **276**–**278** exhibited moderate anti-inflammatory activity by inhibiting LPS-induced NO production, with IC_50_ values of 16.69, 15.87, and 30.48 µM, respectively [76].

A known compound, (+)-(5*R*,5′*R*)-3,3′-methylenebistetronic acid (**279**), was isolated from the mangrove endophytic fungus *Penicillium crustosum* SCNU-F0006. It exhibited inhibitory activity against human pathogenic bacteria and plant pathogenic fungi, including *Pseudomonas aeruginosa* (MIC = 0.5 mg/mL), *Salmonella typhimurium* (MIC = 1.0 mg/mL), *Fusarium oxysporum* (MIC = 0.25 mg/mL), and *Penicillium italicum* (MIC = 0.25 mg/mL) [77]. Two known compounds, butyrolactone I (**280**) and polybotrin (**281**), were isolated from the mangrove-derived fungus *Penicillium* sp. SCSIO 41411. Compound **280** exhibited DPPH radical scavenging activity with an EC_50_ of 16.21 µg/mL. Additionally, compounds **280** and **281** showed weak inhibitory activity against PDE4, with inhibition rates of 29.10% and 26.22%, respectively [8]. A new lactone compound, (*E*)-3-[5-(hydroxymethyl)furan-2-yl-methylene]benzofuran-2(3*H*)-one (**282**), was isolated from the mangrove endophytic fungus *Xylaria arbuscula* QYF [35]. Two new compounds, littoreanoids E-F (**283-284**), were isolated from the mangrove endophytic fungus *Penicillium* sp. HLLG-122. Compound **284** exhibited anti-inflammatory activity with an IC_50_ value of 30.41 µM [78]. Nine new compounds 13-(*R*)-(2-hydroxyethyl)sulfinylmairetolide F (**285**), 13-(*S*)-(2-hydroxyethyl)sulfinylmairetolide F (**286**), 2β,10α,13-trihydroxyeremophil-7(11)-en-12,8β-olide (**287**), 1β,3α,10α-trihydroxyeremophil-7(11)-en-12,8β-olide (**288**), 1β,3α,10α,13-tetrahydroxyeremophil-7(11)-en-12,8β-olide (**289**), 1β,3β,10α,13-tetrahydroxyeremophil-7(11)-en-12,8β-olide (**290**), 1β,2β,10α,13-tetrahydroxyeremophil-7(11)-en-12,8β-olide (**291**), 1-oxo-10α-hydroxyeremophil-7(11)-en-12,8β-olide (**295**), 2-oxo-10α,13-dihydroxyeremophil-7(11)-en-12,8β-olide (**296**), and nine known compounds, mairetolides F-G (**292**–**293**), 13-hydroxymairetolide F (**294**), xylareremophil (**297**), 13-hydroxyxylareremophil (**298**), 2-oxo-eremophil-1(10),7(11),8-trien-12,8-olide (**299**), 2α,13-dihydroxymairetolide A (**300**), mairetolide B (**301**), and eremophil-1(10),7(11),8-trien-12,8-olide-15-oic acid (**302**), were isolated from the mangrove-derived fungus TBRC-BCC 64093. Compounds **285** and **294** exhibited weak cytotoxicity against the Vero (African green monkey kidney) cell line, with IC_50_ values of 49.44 and 186.09 µM, respectively [12]. Two new compounds, citreoviridin H (**303**) and citreoviridin I (**304**), were isolated from the mangrove endophytic fungus *Penicillium* sp. BJR-P2 [26]. Six known compounds, verrucosidinol (**305**), methyl verrucosidinol (**306**), verrucosidinol acetate (**307**), normethylverrucosidin (**308**), verrucosidin (**309**), and penicyrone A (**310**), were isolated from the mangrove endophytic fungus *Penicillium polonicum* H175 [66].

One new compound 2,3-dihydro-2-hydroxyvertinolide (**311**), and two known compounds, 5-hydroxyvertinolide (**312**) and vertinolide (**313**), were isolated from the mangrove endophytic fungus *Trichoderma* sp. FM652. Compound **311** significantly inhibited TNF-α-induced NF-κB activation with an IC_50_ value of 13.83 µM [70]. A known compound, (*R*)-striatisporolide A (**314**), was isolated from the mangrove endophytic fungus *Eupenicillium* sp. [79]. Three known compounds, (4*S*,5*S*,11*R*)-iso-cladospolide B (**315**), (4*S*,5*S*,11*S*)-iso-cladospolide B (**316**), and (4*R*,5*S*,11*R*)-iso-cladospolide B (**317**), were isolated from the mangrove endophytic fungus *Cladosporium* sp. HNWSW-1 [80]. 

Three new compounds, qinlactones A-C (**318**–**320**), were isolated from the mangrove endophytic fungus *Streptomyces qinglanensis* 172205. Compounds **318**–**319** exhibited weak cytotoxic activity against the human breast cancer cell line MCF-7 and the human cervical cancer cell line HeLa, with IC_50_ values ranging from 129 to 207 µM [81]. A known compound, iso-cladospolide B (**321**), was isolated from the mangrove endophytic fungus *Cladosporium oxysporum* HDN13-314. It exhibited antibacterial activity against multiple pathogens, including *Cytospora mandshurica* Miura, *Colletotrichum gloeosporioides*, *Bipolaris sorokiniana*, *Fusarium oxysporum* f. sp. *cucumerinum*, *Edwardsiella tarda*, and *Edwardsiella ictaluri*, with MIC values ranging from 35.09 to 140.35 μM [75]. A new compound, (4*S*,5*S*,6*S*,7*R*)-4-(3-chloro-1,2-dihydroxybutyl)-butyrolactone (**322**), was isolated from the mangrove endophytic fungus *Neofusicoccum parvum* Y2NBKZG1016. At concentrations ≥ 6.25 µM, it showed weak anti-inflammatory activity (NO inhibition), with a maximum inhibition rate of 28.9% [82]. Four new compounds, penipyrols C-F (**323**–**326**), were isolated from the mangrove-derived fungus *Penicillium* sp. HDN-11-131. These compounds feature a rare skeleton in which a γ-butyrolactone is linked via a double bond to an α-pyrrole ring. At 10 µM, compound **323** induced pancreatic β-cell regeneration in zebrafish (45.20 ± 2.359%), exceeding the effect of the positive control prednisolone (39.86 ± 1.773%), indicating promising anti-diabetic potential [83]. Two known compounds, asperteretal G (**327**) and 3-(2-hydroxypropyl)-4-(hexa-2*E*,4*E*-dien-6-yl)furan-2(5*H*)-one (**328**), were isolated from the mangrove sediment-derived fungus *Trichoderma harzianum* SCSIO 41051. Compound **327** exhibited moderate inhibitory activity against acetylcholinesterase (AChE) with an IC_50_ of 2.49 µM and against pancreatic lipase (PL) with an IC_50_ of 2.34 µM. Molecular docking studies indicated interactions between compound **327** and the AChE protein [22]. Four new compounds, asperbutenolides B-C (**329**–**330**) and asperbutenolides E-F (**331**–**332**), along with ten known compounds, butyrolactone III (**333**), (+)-3′,3′-di-(dimethylallyl)-butyrolactone II (**334**), 3-hydroxy-5-(4-hydroxybenzyl)-4-(4-hydroxyphenyl)furan-2(5*H*)-one (**335**), butyrolactone II (**336**), versicolactone B (**337**), asperlide B (**338**), 7″*R*-methoxy-8″*S*-hydroxy-aspernolide E (**339**), asperlide A (**340**), butyrolactone IV (**341**), and aspernolide E (**342**), were isolated from the mangrove-derived fungus *Aspergillus terreus* SCAU011. Compounds **331** and **336** showed COX-2 inhibitory activity superior to the positive control celecoxib. Compounds **334** and **335** exhibited significant α-glucosidase inhibitory activity with IC_50_ values of 56.1 and 12.9 µM, respectively. Meanwhile, compounds **329**, **333**–**336**, and **340**–**342** demonstrated antioxidant activity similar to or better than the positive control curcumin, with IC_50_ values ranging from 0.7 to 23.3 µM. Compounds **334** and **342** showed moderate antibacterial activity against *Staphylococcus aureus*, with IC_50_ values of 17.4 and 36.6 µM, respectively [84]. A known compound, xenofuranone B (**343**), was isolated from the mangrove endophytic fungus *Phyllosticta capitalensis* [17]. Two new compounds, (8″*S*,9′)-dihydroxy-dihydrobutyrolactone I (**344**) and asperbutenolide A (**345**), were isolated from the mangrove endophytic fungus *Aspergillus terreus* SCAU011. At 20 µM, compounds **344** and **345** inhibited cyclooxygenase-2 (COX-2) by 91.8% and 100%, respectively. Compound **345** also exhibited α-glucosidase inhibitory activity (IC_50_ = 10.5 µM) and antibacterial effects against *Staphylococcus aureus* and *Vibrio splendidus*, with IC_50_ values of 1.3 and 3.7 µM, respectively [85]. Two new compounds, (±)-isoepicolactone (±)-**346**, and two known compounds, aepicoccone F (**347**) and 4,5,6-trihydroxy-7-methylphthalide (**348**), were isolated from the mangrove endophytic fungus *Epicoccum nigrum* SCNU-F0002. Compounds (+)-**346** and (−)-**346** showed weak inhibitory activity against COX-2, with inhibition rates of 28.8% and 31.2%, respectively [86].

Two known compounds, 3-(2,6-dihydroxyphenyl)-4-hydroxy-6-methyl-isobenzofuran-1(3*H*)-one (**349**) and 3-(2-deoxy-β-erythro-pentofuranosyl)-6-hydroxy-2*H*-pyran-2-one (**350**), were isolated from the co-culture fermentation products of two mangrove endophytic fungi, *Phomopsis asparagi* DHS-48 and *Phomopsis* sp. DHS-11 [43]. One new compound, embeurekol D (**351**), and one known compound, embeurekol C (**352**), were isolated from the mangrove-derived fungus *Penicillium* sp. SCSIO 41411. The absolute configurations of **351** and **352** were determined by Mosher’s ester method and ECD calculations. At a concentration of 10 µM, compounds **351** and **352** exhibited weak inhibitory activity against PDE4, with inhibition rates of 18.62% and 14.95%, respectively [8]. Using an OSMAC strategy, Liu et al. identified a known compound, astalaminoid C (**353**), from the mangrove-derived fungus *Penicillium* sp. HDN15-312. It exhibited moderate DPPH radical scavenging activity with an IC_50_ value of 32.11 μM [40]. A known compound, 4-(hydroxymethyl)-5,7-dimethoxy-6-methylisobenzofuran-1(3*H*)-one (**354**), was isolated from the mangrove endophytic fungus *Aspergillus* sp. GXNU-Y85 [87]. Two new compounds, pestalotiophthalides A-B (**355–356**), and four known compounds, 5,7-dimethoxy-4,6-dimethylisobenzofuran-1(3*H*)-one (**357**), 7-hydroxy-5-methoxy-4,6-dimethylisobenzofuran-1(3*H*)-one (**358**), 6-(hydroxymethyl)-5,7-dimethoxy-4-methylisobenzofuran-1(3*H*)-one (**359**), and 4-(hydroxymethyl)-5,7-dimethoxy-6-methylisobenzofuran-1(3*H*)-one (**360**), were isolated from the mangrove endophytic fungus *Pestalotiopsis* sp. SAS4 [88]. One new compound, 3-hydroxyepicoccone B (**361**), and three known compounds, 4,6-dihydroxy-5-methoxy-7-methylphthalide (**362**), 4,5,6-trihydroxy-7-methyl-3*H*-isobenzofuran-1-one (**363**), and sparalide C (**364**), were isolated from the mangrove endophytic fungus *Epicoccum nigrum* MLY-3. At 10 µg/mL, compounds **361** and **363** exhibited DPPH radical scavenging activity with IC_50_ values of 29.3 and 16.5 µM, respectively, and ABTS radical scavenging activity with IC_50_ values of 23.7 and 23.3 µM, respectively, outperforming the positive control acarbose (IC_50_ = 33.6 ± 0.8 µM) [89]. Two known compounds, pestaphthalide A (**365**) and (*S*)-3-[(*S*)-1-hydroxyethyl]-5,7-dimethoxy-6-methylisobenzofuran-1(3*H*)-one (**366**), were isolated from the mangrove endophytic fungus *Botryosphaeria ramose*. Compounds **365** and **366** exhibited inhibitory activity against *Penicillium italicum*, with IC_50_ values of 223.21 and 99.21 µM, respectively, which were stronger than the positive control triadimefon (IC_50_ = 170.65 µM) [28].

A known compound, dimethoxyphtalide (**367**), was isolated from the mangrove-derived fungus *Cytospora* sp. [30]. Five new compounds, (±)-epicoccone C (±**368**), epicoccone D (**369**), epicoccone E (**370**), epicolactone A (**371**), and one known compound, epicolactone (**372**), were isolated from the mangrove endophytic fungus *Epicoccum nigrum* SCNU-F0002. Compounds (+)-**368** and **370** exhibited strong α-glucosidase inhibitory activity with IC_50_ values of 43.2 and 53.2 µM, respectively, stronger than the positive control acarbose. Compounds (-)-**368**, **369**, and **371** showed moderate inhibitory activity, with IC_50_ values ranging from 130.2 to 252.4 µM. In addition, compounds (±)-**368** demonstrated antioxidant activity stronger than the positive controls gallic acid and vitamin C, with IC_50_ values of 11.1 and 10.2 µM, respectively [90]. Four new compounds, trichoderolides C-F (**373**–**376**), and one known compound, (3*R*,5*R*)-harzialactone A (**377**), were isolated from the mangrove endophytic fungus *Trichoderma erinaceum* F1-1 [73].

### 2.6. Azaphilones

**Figure 6:**
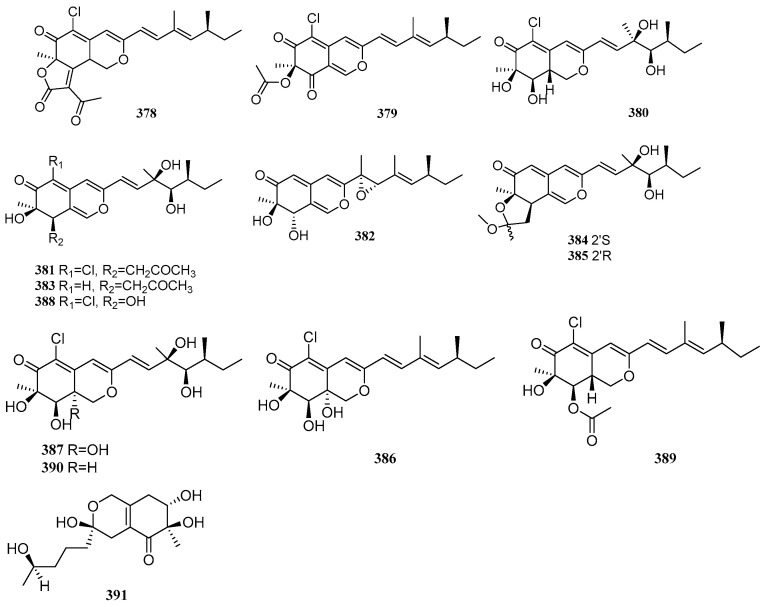
Azaphilones in polyketide compounds of mangrove fungi.

Azaphilones are a class of fungal polyketides characterized by a pyranoquinone bicyclic core. Between 2020 and 2025, a total of 14 azaphilone compounds were isolated and identified from mangrove-derived fungi. Their structures are shown in Figure 6.

A compound, 5-chloroisorotiorin (**378**), was isolated from the mangrove-derived fungus *Phomopsis* sp. TJM1-5 [91]. Two compounds, (+)-sclerotiorin (**379**) and geumsanol G (**380**), were isolated from the mangrove endophytic fungus *Penicillium sclerotiorum* SCNU-F0040 [92]. Seven new compounds, peniazaphilones C-I (**381**–**387**), along with three known compounds geumsanol F (**388**), isochromophilone IV (**389**), and isochromophilone WB (**390**), were isolated from the mangrove endophytic fungus *Penicillium sclerotiorum* ZJHJJ-18. Compounds **389** and **390** inhibited LPS-induced NO release with IC_50_ values of 17.64 and 4.71 µM, respectively, demonstrating stronger activity than the positive control indomethacin (IC_50_ = 35.27 µM) [93]. Xu et al. identified a compound, pestalotiopsol A (**391**), from the mangrove-derived fungus *Pestalotiopsis* sp. HHL101 [20].

### 2.7. Others

**Figure 7:**
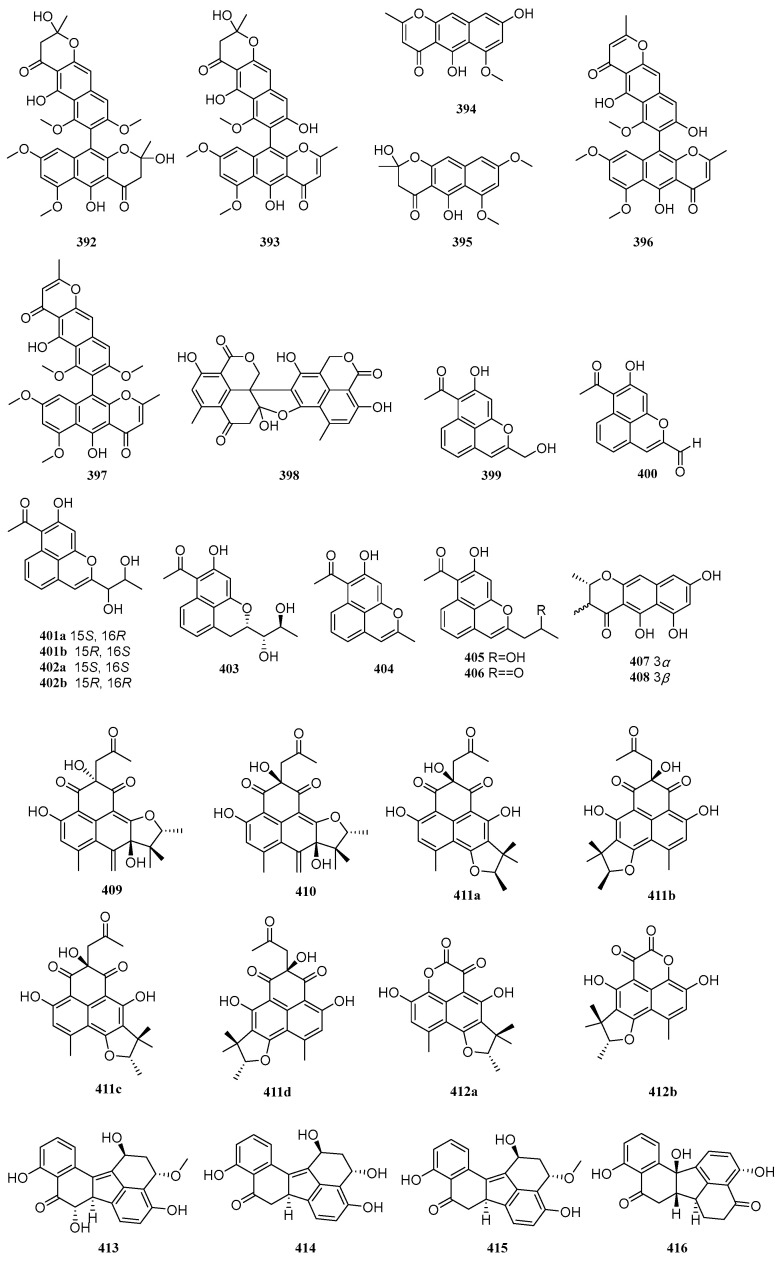

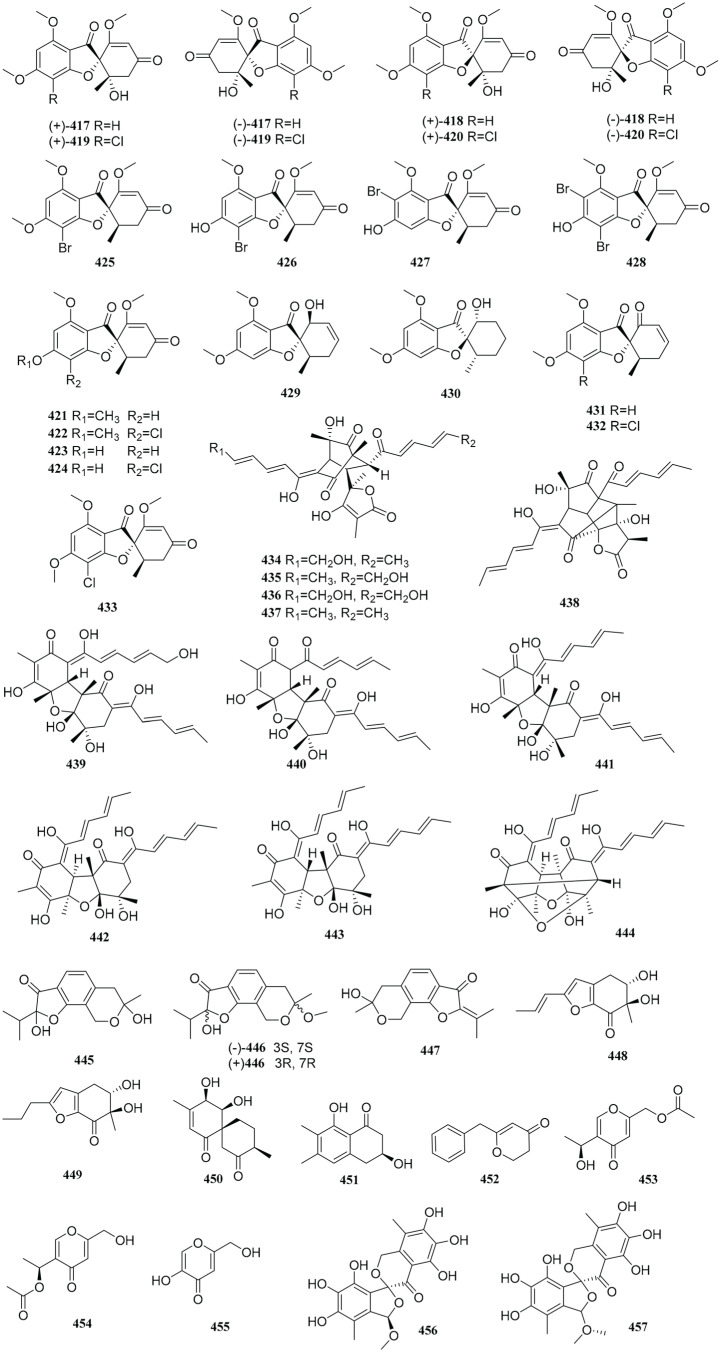
Others in polyketide compounds of mangrove fungi.

Polyketides exhibit diverse structural types beyond those mentioned above, including various other skeletons. Between 2020 and 2025, a total of 66 other polyketide compounds were isolated and identified from mangrove-derived fungi. Their structures are shown in Figure 7.

Six known compounds, aurasperone B (**392**), aurasperone F (**393**), TMC-256A1 (**394**), fonsecin B (**395**), dianhydroaurasperone C (**396**), and aurasperone A (**397**), were isolated from three mangrove-derived fungi, *Aspergillus* sp. IQ-503, *Aspergillus* sp. IQ-548, and *Talaromyces* sp. I-567. Compounds **392**–**394** exhibited inhibitory effects on bacterial growth, with IC_50_ values ranging from 6.9 to 9.9 µg/mL. Through in vitro evaluation of molecular interactions with the *Acinetobacter baumannii* filamenting temperature-sensitive mutant Z (AbFtsZ) protease to identify anti-*A. baumannii* agents, it was found that compounds **392**, **393**, and **395** enhanced AbFtsZ activity under interaction, whereas compound **394**, as the sole inhibitor of AbFtsZ, suppressed bacterial growth [94]. A known compound, bacillisporin C (**398**), was isolated from the mangrove-derived fungus *Talaromyces* sp. WHUF0362 [11]. Five new compounds, RM18c-RM18g (**399**–**403**), and three known compounds, RM18b (**404**), wailupemycin K (**405**), and RM18 (**406**), were isolated from the mangrove endophytic fungus *Streptomyces* sp. SCSIO 40069. Among these, compounds **401** and **402** constitute a pair of racemates. Compounds **399**–**401**, **402b**, and **406** exhibited antibacterial activity against *Acinetobacter baumannii* ATCC 19606, *Vibrio alginolyticus* ATCC 13214, *Staphylococcus aureus* ATCC 29213, *Klebsiella pneumonia* ATCC 13883, and *Micrococcus luteus* SCSIO ML01, with MIC values ranging from 8 to 64 μg/mL [95]. Two new compounds, peninaphones A (**407**) and B (**408**), were isolated from the mangrove endophytic fungus *Penicillium* sp. HK1-22. Compounds **407**–**408** showed weak antibacterial activity against *Staphylococcus aureus*, with inhibition zone diameters ranging from 10.4 to 21.0 mm [96]. Four new compounds, aceneoherqueinones A and B (**409**–**410**), (+)-aceatrovenetinone A (**411a**), and (+)-aceatrovenetinone B (**411d**), along with four known compounds, (-)-aceatrovenetinone A (**411b**), (-)-aceatrovenetinone B (**411c**), (-)-scleroderolide (**412a**), and (+)-scleroderolide (**412b**), were isolated from the mangrove endophytic fungus *Penicillium herquei* MA-370. Compounds **409** and **410** inhibited angiotensin-converting enzyme (ACE) with IC_50_ values of 3.10 and 11.28 µM, respectively. Molecular docking analysis elucidated the intermolecular interactions and potential binding sites of **409** and **410** with ACE, indicating that compound **409** binds favorably via hydrogen interactions with residues Ala261, Gln618, Trp621, and Asn624, while compound **410** interacts with residues Asp358 and Tyr360 [97]. One new compound, guhypoxylonol A (**413**), and three known compounds, hypoxylonol C (**414**), hypoxylonol B (**415**), and daldinone C (**416**), were isolated from the mangrove endophytic fungus *Aspergillus* sp. GXNU-Y45. Compounds **413** and **415** inhibited LPS-induced NO production with IC_50_ values of 14.42 and 21.05 µM, respectively, compared to the positive control dexamethasone (IC_50_ = 16.12 µM) [98].

Zou et al. activated silent biosynthetic genes by modifying culture medium components and adding sodium bromide/sodium chloride, leading to the isolation and identification of 12 new compounds, (±)-6′-hydroxy-7-dechlorogriseofulvin [(±)-**417**], (±)-6′-hydroxy-7-dechloroepigriseofulvin [(±)-**418**], (+)-6′-hydroxygriseofulvin [(+)-**419**], (±)-6′-hydroxyepigriseofulvin [(±)-**420**], 6-*O*-desmethyl-7-bromogriseofulvin (**426**), 5-bromo-6-*O*-desmethyl-7-dechlorogriseofulvin (**427**), 5,7-dibromo-6-*O*-desmethylgriseofulvin (**428**), 3′,4′-dihydroeupenigriseofulvin (**430**), 4′-demethoxy-7-dechloroisogriseofulvin (**431**), along with two new natural products, 7-bromogriseofulvin (**425**) and 4′-demethoxyisogriseofulvin (**432**), and six known analogs, (−)-6′-hydroxygriseofulvin [(−)-**419**], 7-dechlorogriseofulvin (**421**), griseofulvin (**422**), 6-*O*-desmethyl-7-dechlorogriseofulvin (**423**), 6-*O*-desmethylgriseofulvin (**424**), and eupenigriseofulvin (**429**), from the mangrove-derived fungus *Nigrospora* sp. QQYB1. Compounds **422** and **425** exhibited significant antifungal activity against *Colletotrichum truncatum*, *Microsporum gypseum*, and *Trichophyton mentagrophytes*, with inhibition zone diameters ranging from 28 to 41 mm (10 μg/disk). Structure-activity relationship studies revealed that substitutions at C-6, C-7, and C-6′, as well as the positions of carbonyl groups and double bonds, significantly influenced antifungal potency. Comparison of compounds **422**–**424** and **429**–**431** (or **425**–**428**) showed that a 6-methyl group enhanced antifungal activity, while substitution with a 6-hydroxyl group markedly reduced activity. Evaluation of compounds **421**–**422** and **425** indicated that halogen atoms at C-7 contributed to antifungal efficacy, with bromine substitution at C-7 causing substantial changes in activity. Furthermore, comparing compounds **417**–**420** with **422** demonstrated that hydroxylation at C-6′ significantly diminished antifungal activity [99]. A known compound griseofulvin (**433**), was isolated from the mangrove endophytic fungus *Arthrinium* sp. SCSIO 41306. It inhibited LPS-induced NF-κB activation in RAW264.7 macrophages with an IC_50_ value of 22.21 µM and showed no significant cytotoxicity in bone marrow-derived macrophages (BMMs) [100]. Li et al. identified three new compounds, 14-hydroxybislongiquinolide (**434**), 20-hydroxybislongiquinolide (**435**), and 14,20-dihydroxybislongiquinolide (**436**), along with four known compounds, bislongiquinolide (**437**), bisorbicillinolide (**438**), saturnispol B (**439**), and bisvertinolone (**440**), from the mangrove-derived fungus *Trichoderma reesei* SCNU-F0042. Compound **435** exhibited moderate SARS-CoV-2 inhibitory activity with an EC_50_ value of 29.0 µM [101]. A known compound bisvertinol (**441**), was isolated from the mangrove endophytic fungus *Hypocrea jecorina* H8 [102].

Three known compounds, isobisvertinol (**442**), bisvertinol (**443**), and trichodimerol (**444**), were isolated from the mangrove endophytic fungus *Trichoderma* sp. FM652. Compounds **442** and **443** inhibited TNF-α-induced NF-κB pathway activation with IC_50_ values of 24.40 and 14.63 µM, respectively. Compound **444** showed moderate antibacterial activity against *Staphylococcus aureus* and methicillin-resistant *S. aureus* with an MIC value of 40.32 µM [70]. Three new compounds, asperisocoumarin H (**445**) and (±)-asperisocoumarin I [(±)-**446**], and one known compound, pergillin (**447**), were isolated from the mangrove endophytic fungus *Aspergillus* sp. 085242. Compound **447** exhibited α-glucosidase inhibitory activity with an IC_50_ value of 428.1 µM, stronger than the positive control acarbose (IC_50_ = 725.1 µM) [37]. Using an OSMAC strategy, Liu et al. identified two new compounds, furantides A-B (**448**–**449**), from the mangrove-derived fungus *Penicillium* sp. HDN15-312 [40]. A known compound, penicyclone A (**450**), was isolated from the mangrove sediment-derived fungus *Penicillium* sp. N-5. It was evaluated for cytotoxicity against SNB-19, MDA-MB-231, MDA-MB-435, and HCT-116 cell lines but showed no cytotoxic activity [103]. A known compound (3*S*)-3,8-dihydroxy-6,7-dimethyl-α-tetralone (**451**), was isolated from the mangrove endophytic fungus *Daldinia eschscholzii* MCZ-18. It exhibited broad-spectrum antibacterial activity against five pathogens, Enterococcus *faecalis*, methicillin-resistant *Staphylococcus aureus*, *Escherichia coli*, *Pseudomonas aeruginosa*, and *Candida albicans*, with IC_50_ values ranging from 6.25 to 50 µM [9]. A known compound, 2-benzylpyrone (**452**), was isolated from the mangrove endophytic fungus *Mycosphaerella* sp. L3A1 [50]. Two new compounds, phomasparapyrones A (**453**) and B (**454**), and one known compound, kojic acid (**455**), were isolated from the mangrove endophytic fungus *Phomopsis asparagi* LSLYZ-87. Compound **454** showed dose-dependent inhibition of LPS-induced NO accumulation in BV-2 cells at 30, 40, and 50 µM, with no cytotoxicity observed at 50.0 µM [104]. Two new compounds, aspermicrone B (**456**) and aspermicrone C (**457**), were isolated from the mangrove endophytic fungus *Epicoccum nigrum* SCNU-F0002 [86].

## 3. Conclusions

Mangrove forests, as unique marine-terrestrial ecotones, are subjected to extreme environmental conditions. Their endophytic fungi have evolved distinctive secondary metabolites to adapt to these complex ecological niches. In recent years, research on secondary metabolites from mangrove-derived fungi has revealed their multifaceted value in drug discovery, agricultural applications, and ecological conservation, establishing this area as a hotspot at the intersection of natural product chemistry and synthetic biology.

The chemical diversity of metabolites from mangrove-derived fungi is remarkable, primarily encompassing structural classes such as polyketides, alkaloids, terpenoids, and peptides. Over 1400 new compounds have been discovered from more than 300 fungal strains belonging to over 70 genera isolated from mangrove ecosystems. These structures include polyketides, terpenoids, alkaloids, peptides, among others, with over 40% exhibiting biological activities such as cytotoxicity, antimicrobial, antiviral, anti-inflammatory, and neuroprotective effects [2]. As a major metabolite class from these fungi, the continuous discovery of polyketides consistently expands the structural libraries available for medicinal chemistry.

This review covers 457 polyketide metabolites isolated from mangrove-derived fungi between January 2020 and February 2025, comprising 176 new compounds (including 5 featuring novel skeletons) and 5 new natural products. Among these, 201 compounds (44.0%) demonstrated biological activities, primarily antioxidant, anti-inflammatory, and antimicrobial effects, indicating their potential for pharmaceutical research, agricultural applications, and cross-disciplinary development.

**Figure 8:**
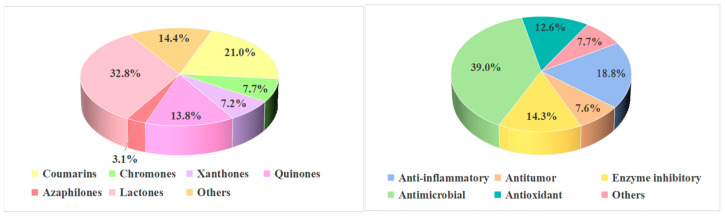
Compounds types and bioactivity distribution of polyketides from mangrove-derived fungi.

**Figure 9:**
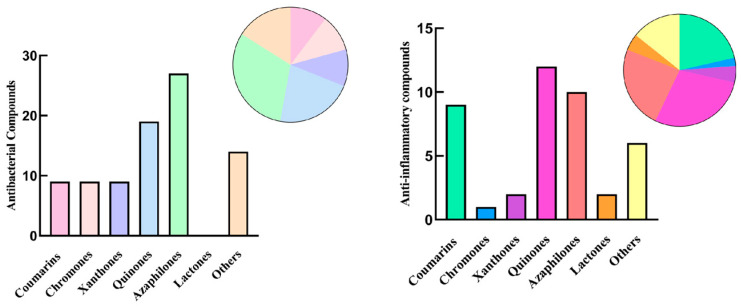
Distribution of antibacterial and anti-inflammatory compound types.

The polyketide constituents from mangrove-derived fungi are categorized into seven major structural classes, 96 coumarins and isocoumarins, 35 chromones, 33 xanthones, 63 quinones, 150 lactones, 14 azaphilones, and 66 other polyketide-type compounds. Lactones represent the predominant class (32.8%), followed by coumarins and isocoumarins (21.0%), and other structural types (14.4%) (Figure 8). Antimicrobial and anti-inflammatory activities are the most prominent biological properties observed. Specifically, 87 compounds (39.0% of active compounds) exhibited antimicrobial activity, while 42 compounds (18.8% of active compounds) showed anti-inflammatory activity. The most active structural types were coumarins and isocoumarins, quinones, and azaphilones. The distribution of active compounds by structural class is summarized in Figure 9.

The unique nature of the mangrove ecosystem has driven metabolic adaptive evolution in mangrove-derived fungi, promoting the synthesis of structurally novel and biologically significant secondary metabolites, thereby establishing them as an important source of new active natural products. According to statistics, mangrove fungi exhibit a 2.3–4.1 fold higher abundance of polyketide synthase (PKS) and nonribosomal peptide synthetase (NRPS) gene clusters compared to terrestrial fungi, highlighting their prominent role as a source of lead compounds. With the deepening integration of genomics and metabolomics, systematic exploration using chemical and molecular biological methods, including chemical epigenetic modification, co-cultivation, the OSMAC approach, and genome mining, can be employed to discover more bioactive substances. These findings not only provide novel chemical entities for drug synthesis and candidate molecules for marine drug development but also offer a theoretical foundation and material basis for the conservation and sustainable utilization of mangrove resources.

## Data Availability

Not applicable.
